# Feeling good, approaching the positive

**DOI:** 10.3389/fpsyg.2024.1491612

**Published:** 2024-12-18

**Authors:** Kristina Kobrock, Johannes Solzbacher, Nicole Gotzner, Peter König

**Affiliations:** ^1^Institute of Cognitive Science, University of Osnabrück, Osnabrück, Germany; ^2^Institute of Neurophysiology & Pathophysiology, University Medical Center Hamburg-Eppendorf, Hamburg, Germany

**Keywords:** approach-avoidance behavior, affect, Approach-Avoidance Bias (AAB), mood induction, emotional priming, PANAS

## Abstract

**Introduction:**

Approach and avoidance behaviors have been extensively studied in cognitive science as a fundamental aspect of human motivation and decision-making. The Approach-Avoidance Bias (AAB) refers to the tendency to approach positive stimuli faster than negative stimuli and to avoid negative stimuli faster than positive ones. Affect and arousal in involved individuals are assumed to play a crucial role in the AAB but many questions in that regard remain open. With this in mind, the present study aimed to examine the impact of positive and negative mood on the AAB.

**Method:**

To achieve this goal, we conducted an experiment where participants watched either positive or negative videos prior to performing an approach-avoidance task.

**Results:**

We have not been able to confirm our preregistered hypothesis that mood induction moderates the AAB. Instead, our results suggest that an AAB can be robustly shown after both the positive and the negative intervention. Positive Affect Negative Affect Schedule (PANAS) results show that the participants' affective state was influenced by the mood intervention only in the form of increased emotional intensity. Participants did not self-report a change in mood valence that corresponds to the valence of the video primes. However, the behavioral data shows that after watching a positive video, participants are faster in approaching positive stimuli than negative stimuli. At the same time, we do not find a similar effect after the negative intervention.

**Discussion:**

These findings suggest that positive and negative affect might play an important role in shaping the AAB that is modulated by stimulus valence. This provides new potential insights into the underlying mechanisms of human motivation and decision-making. Specifically, we argue for potential differences between attention and reaction toward a valenced stimulus.

## 1 Introduction

Approach-avoidance behavior refers to the conflicting tendencies of an individual to approach or avoid a stimulus or situation. It is thought to arise from a complex interplay of biological, psychological, and social factors and is relevant in many real-world situations, such as addiction (Booth et al., 2018; Ernst et al., 2014), phobia (Rinck and Becker, 2007), and depression (Radke et al., 2014). Thus, understanding its mechanisms is not only relevant for scientific purposes but might also be helpful in developing effective treatments and interventions for people who are struggling with these issues and where maladaptive approach or avoidance behaviors can lead to significant negative consequences (see Beevers et al., 2015; Luo et al., 2015; Wiers et al., 2015, 2013b for promising approaches).

One way to assess an individual's bias to approach or avoid certain stimuli rather than others is what is called the Approach-Avoidance Task (AAT).^1^ In this setup, participants respond to stimuli by approaching or avoiding them, for example, by pushing and pulling a joystick or by pressing different buttons (Solzbacher et al., 2022). The paradigm developed from a very basic manual setup (Solarz, 1960), to a digital setup implementing zoom-effects of the stimuli (e.g. Rinck and Becker, 2007; Rougier et al., 2018) up to a highly variable setup branching out even into the integration of imagery (Fridland et al., 2022) or whole-body-movements in virtual reality (Eder et al., 2021; Eiler et al., 2019).

In experiments using the AAT, a number of different Approach-Avoidance Biases (AABs) have been shown to be present in different groups of individuals. Healthy adults show a bias to generally approach positive stimuli faster than they avoid them and avoid generally negative stimuli faster than they approach them (e.g., Solzbacher et al., 2022). The same holds for approaching chocolate (Dickson et al., 2016) or food in general (e.g., Booth et al., 2018). Individuals with a fear of spiders exhibit an avoidance bias toward stimuli showing spiders (e.g., Rinck and Becker, 2007), socially anxious individuals avoid smiling and angry faces faster than controls (Heuer et al., 2007), smokers (but not ex-smokers) exhibit an approach bias toward smoking cues (Wiers et al., 2013a), the same holds for users of heroin (Zhou et al., 2012), alcohol (Ernst et al., 2014; Wiers et al., 2014; Gladwin et al., 2013) and cannabis (Cousijn et al., 2011). Additionally, a correlation between drinking behavior and the alcohol-approach bias was found (Peeters et al., 2012), suggesting that cognitive biases of this kind are not all-or-nothing biases but instead follow a certain scalability in the form of a “the more—the stronger”-principle. Altogether, research has shown that different psychological dispositions yield different biases, but most of them represent a tendency to approach what one likes, wants, or needs and to avoid the scary or disgusting.

While the existence of AABs as such is undisputed, their mechanisms are still unclear, with three main options at hand. First, embodied approaches to the AAB put their focus on the involved movements and argue that the bodily (physical) action of approaching or avoiding (i.e., moving the arm, moving the body, etc.) plays a crucial role in the process (e.g. Chen and Bargh, 1999; Solzbacher et al., 2022; Fridland and Wiers, 2018; Eder et al., 2021). Second, evaluative coding accounts propose that approach-avoidance compatibility effects are mainly driven by automatic associations between stimuli and evaluative responses. While early accounts of event-coding do not mention the involvement of affect in the process (Hommel et al., 2001), later versions propose an “emotionally enriched” version of the account (Lavender and Hommel, 2007). Third, some have proposed accounts based on distance change that focus on the perceived spatial relationship between the individual and the stimulus (Seibt et al., 2008; Krieglmeyer and Deutsch, 2010; Bouman et al., 2022). And while all of those models have their fair points, regarding the role of affect, the proposed theories often focus on stimulus valence and do not consider the affective states of involved subjects. Yet, it seems evident that a crucial aspect of figuring out which group of subjects yields which effect is the particular affective predisposition that the specific group shares with regard to the stimuli. If they fear the kind of stimulus shown (e.g., spiders), they share the tendency to avoid those rather than to approach them, if they strive for the kind of stimulus shown (e.g., subjects suffering from substance use disorder seeing stimuli depicting the substance), they share the tendency to faster approach those than to avoid them. Particularly interesting in that regard are results observed in individuals suffering from depression. While healthy individuals usually exhibit an approach bias toward happy and an avoidance bias toward angry faces (e.g. Volman et al., 2011) and voices see (Ikeda, 2023a), individuals suffering from depression show no such bias at all and react to angry and happy faces equally (e.g., Radke et al., 2014). One interpretation of those findings could be that a particular form of negative affective predisposition, as present in depression, countermeasures the natural disposition to approach the good and to avoid the bad, as we see it in healthy individuals. While previous studies and theories have examined the connections between stimulus valence and arm movement (e.g., Chen and Bargh, 1999; Krieglmeyer et al., 2010), between stimulus evaluation and perceived stimulus valence (e.g., Neumann and Strack, 2000), and between task setup and stimulus valence (e.g., Krieglmeyer and Deutsch, 2010), given the preceding discussions, the research on stimulus valence is extensive but also an individual's current mood or affective state could plausibly influence the described compatibility effect.

Thus, the starting point of our research is the assumption that the affective states of the subjects might play a role in the AAB. For our purposes, we define affect as an umbrella term that subsumes short-term affective states like an emotion, but also long-term affective states such as mood. While many scholars agree on the distinction between emotion and mood, there is no consensus on what exactly are the distinguishing properties (Beedie et al., 2005; Gomez et al., 2009). Here, we adopt the convention that emotions are about objects and stimuli that are present, whereas mood can be more global and sustained (Gomez et al., 2009; Frijda, 1994). Regarding the AAT, the stimuli, which carry a particular affective valence, can elicit an emotion in the participants. Mood, on the other hand, is the affective state of an individual that can go beyond the (short) time in which a stimulus is present.

While the role of affect in terms of stimulus valence has been vastly investigated in the past (e.g. Phaf et al., 2014), it is far less clear if or how exactly an individual's mood influences their evaluation of or reaction toward an encounter. Related research has investigated the role of individual predispositions in specific situations (see Grèzes et al., 2021 for a study on approach-avoidance behavior being affected by sleep deprivation) or groups (see Krehbiel et al., 2021; Zech et al., 2023 for studies with individuals suffering from eating disorder). The connection between an individual's more general mood and approach-avoidance behavior, however, has not yet been extensively researched. A single paper has studied the influence of mood induction on reaction times in indirect AATs using happy, sad, and neutral faces (Vrijsen et al., 2013). In that study, affective dispositions did not produce different behavioral biases. However, first, it is known that AATs with indirect instructions produce generally weaker biases than AATs with direct instructions (Phaf et al., 2014), and some studies even suggest that conscious affective evaluation of a stimulus is necessary for the effect to be present (Rotteveel and Phaf, 2004). Second, (Vrijsen et al., 2013) tested for the effect between subjects, while it is known that subject-to-subject differences in AATs are quite potent. Third, only female students participated in the study, such that a gender effect cannot be excluded. So, while there has been a study investigating the connection between mood and approach-avoidance behavior, further research in that area seems asked for.

With this paper, we try to shed more light on the connection between this specific dimension of affect (as involved in an individual's mood) and approach-avoidance behavior. To achieve this, we developed a within-subjects Approach-Avoidance Task setup in which we used an intervention aimed at inducing positive and negative mood in subjects in two different sessions, respectively. Before and after the intervention, we let participants self-report their affective state using the Positive Affect Negative Affect Schedule (PANAS) questionnaire (Watson et al., 1988), followed by an Approach-Avoidance Task using a joystick and positive and negative pictures from the International Affective Picture System (IAPS; Lang et al., 1997). We hypothesized that the induction of a specific mood might have an effect on reaction times in an AAT. Specifically, we expected an effect in negative mood facilitating performance in the incongruent condition (i.e., avoiding positive stimuli and approaching negative stimuli) and positive mood facilitating performance in the congruent condition (i.e., approaching positive stimuli and avoiding negative stimuli). One motivation to test this hypothesis came from depression research and reflects the finding that individuals suffering from depression do not show an AAB in the classic sense. If the reason for this was their “negative mood,” this might suggest that negative mood influences reactions in the incongruent condition such that participants show no or a weaker AAB regarding positive and negative stimuli. However, we would like to stress that the main goal of this study was not to shed light on behavior in depression but to shed light on the role of affect in an AAB as exhibited in healthy populations. This study allows us to gain insights into the more general mechanisms of approach and avoidance behavior that are not confined to specific patient groups. Our results can then inform further research in the respective patient groups dealing with either approach biases such as in patients with substance disorders or avoidance biases such as in patients with anxiety or, a combination of reduced approach motivation for positive stimuli and reduced avoidance motivation for negative stimuli as is hypothesized for individuals suffering from depression (e.g. Loijen et al., 2020).

## 2 Material and methods

Our study combines a standard joystick-AAT that uses positive and negative images as stimuli with an intervention in which we showed participants positive or negative videos. This intervention was designed to change the participant's mood (see our preregistration). Before and after the mood induction, the participants were asked to rate their current affective state using the Positive Affect Negative Affect Schedule (PANAS; Watson et al., 1988) questionnaire. The detailed procedure is described below. The experiment was conducted between 11-29-2021 and 02-22-2022. Before the start of data collection, the ethics committee of the University of Osnabrck approved the study. We report how we determined our sample size, all data exclusions, all manipulations, and all measures in the study. This study's design, hypotheses, and analysis plan were preregistered (see https://osf.io/65dzx/). All data and analysis scripts have been made publicly available at the Open Science Framework (OSF) repository and can be accessed at https://osf.io/84th6/. All materials used can be accessed via the original sources (Watson et al., 1988; Lang et al., 1997; Schaefer et al., 2010). The analyses were conducted in R version 4.2.2 (R Core Team, 2022) using the R package brms (Bürkner, 2017).

### 2.1 Participants

A sample of 101 subjects has been recruited via university e-mail lists and postings on the electronic bulletin boards of Osnabrück University and Osnabrück University of Applied Sciences. The sample size was determined based on estimates obtained from previous research. For the AAB, we assumed an effect size of about 50 ms ranging from 0 to 100 ms. Sample sizes in previous research investigating the AAB range from 20 to 100 participants, with most studies having samples between 20 and 50 participants (Phaf et al., 2014). Sample sizes for mood inductions assessed with the PANAS questionnaire range between 20 (Rahimi et al., 2019) and 170 (Peel et al., 2023) participants, according to a literature search.^2^ In the Bayesian framework we use to analyse our data, the detectable effect size does not only depend on the sample size but also on the priors used in the analyses. We report the results of a sensitivity analysis over the priors and which effect sizes we can detect with our choice of priors in the Supplementary material in the section “Prior predictive checks.”

Inclusion criteria were a self-assessed good understanding of the English language and a minimum age of 18 years at the date of participation. Exclusion criteria were applied when subjects did not have normal or corrected to normal vision and hearing as well as a generally healthy condition (no musculoskeletal constraints or problems with the nervous system). Six subjects had to be excluded because of either of the three reasons: (1) no or incomplete adherence to the inclusion and exclusion criteria (one subject), (2) measurement errors (one subject), or (3) data from both sessions could not be collected (four subjects). The final sample of 95 participants comprised 20 male, 75 female, and no with diverse genders and 90 right and five left-handed participants. Their age ranged from 18 to 35, with an average age of 23 (*mean* = 22.95, *sd* = 3.69, *median* = 22). According to the data minimization principles of the General Data Protection Regulation (GDPR), data on racial identity, ethnicity, nativity or immigration history, and socioeconomic status were not collected. Participants received financial compensation of 19 euros or two VP-hours which could later be transferred into course credits for students of Psychology or Cognitive Science. They have been asked to give written informed consent before the start of the experiment. All instructions were given in English.

### 2.2 General apparatus

The experiment was conducted with one participant at a time in a quiet laboratory room. Participants were seated in front of a desk with all devices positioned in front of them, supporting natural and effortless use. The image and video stimuli were presented on a 24” LCD monitor (BenQ XL2420T; BenQ, Taipeh, Taiwan) with a resolution of 1,920 × 1,080 pixels and a refresh rate of 114 Hz. The main experiment interface was programmed in Python (version 3.5.6) to ensure a smooth transition between the emotional video shown before the measurements proper and AAT. Participants listened to the sounds via speakers at a medium loud volume. The light in the room was dimmed to support a good atmosphere for watching the mood induction video and to ensure that the screen had the undivided attention of the participants. Readable instructions and video stimuli were presented in English. The joystick used for the AAT (Logitech Attack TM 3; Logitech, Apples, Switzerland) was placed on the table in front of the participants, and it was directly connected to the computer screen. The reaction times of pushing and pulling movements were recorded via Matlab's Psychtoolbox V3 (Kleiner et al., 2007) (r2017a; MathWorks Company). This implementation was reused from previous studies (Czeszumski et al., 2021; Solzbacher et al., 2022) and only slightly customized to the current purposes.

### 2.3 Stimuli

As preregistered, we used video stimuli as an intervention. As suggested by meta-analyses and a systematic review on this topic, videos are best suited for eliciting emotions or mood in a laboratory setting, especially of negative valence (Gerrards-Hesse et al., 1994; Westermann et al., 1996; Fernández-Aguilar et al., 2019). The assumption that a mood induced with short videos is stable over a period of about 20 min is common in the literature and has been empirically confirmed (see e.g. Tan and Qu, 2015). The AAT that the participants completed after the mood induction procedure lasted about 20 min. For our study, we selected two emotional movie scenes from a database that was composed for emotion research on the basis of participants' ratings (Schaefer et al., 2010). Schaefer et al. (2010) let 364 participants rate 70 movie scenes based on two scales, the PANAS (Watson et al., 1988) and the Differential Emotions Scale (DES, Izard et al., 1974). The authors evaluated the movies based on these ratings and sorted them into six emotional categories: anger, amusement, disgust, fear, sadness, and tenderness. As an objective criterium, we had preregistered to use the scenes with the highest positive affect (PA) score on the DES scale from 1 to 5 for the positive mood induction and the scene with the highest negative affect (NA) score on the DES scale for the negative mood induction. Accordingly, we used “The Dead Poets Society [2]” from the full-colored movie “Dead Poets Society” (Weir, 1989) for the positive intervention. It scored 2.82 on the PA scale and 1.21 on the NA scale, takes 2:40 min, and belongs to the category *tenderness*. For the negative intervention, we used the black and white excerpt “American History X” from “American History X” (Kaye, 1998). It scored 2.04 on the PA scale and 2.73 on the NA scale, takes 1:17 min, and belongs to the category *anger*).^3^ Reducing the full affective spectrum to just one core affect has limitations. But for the sake of a clear operationalization of positive vs. negative intervention, we decided to rely on the PA and NA scores as rated in the validation study by Schaefer et al. (2010).

For the AAT, 88 full-colored images from the International Affective Picture System (IAPS; Lang et al., 1997) were used as stimuli. The IAPS is a collection of images standardized for the study of emotions. They are rated on three dimensions: Valence, arousal, and dominance. While the original validation of these stimuli was almost thirty years ago, a recent systematic review confirms that the IAPS is still the number one choice for eliciting emotions with picture stimuli (Branco et al., 2023). The stimulus set used comprised 44 negative images with a valence rating below 3 and 44 positive images with a valence rating above 7.2. It was identical to the stimuli employed in other studies (Solzbacher et al., 2022; Czeszumski et al., 2021). All pictures were presented in their native resolution of 1,024 × 768 pixels and placed in the middle of the screen on a gray background (RGB values: 182/182/182).

### 2.4 Design and procedure

**Figure 1:**
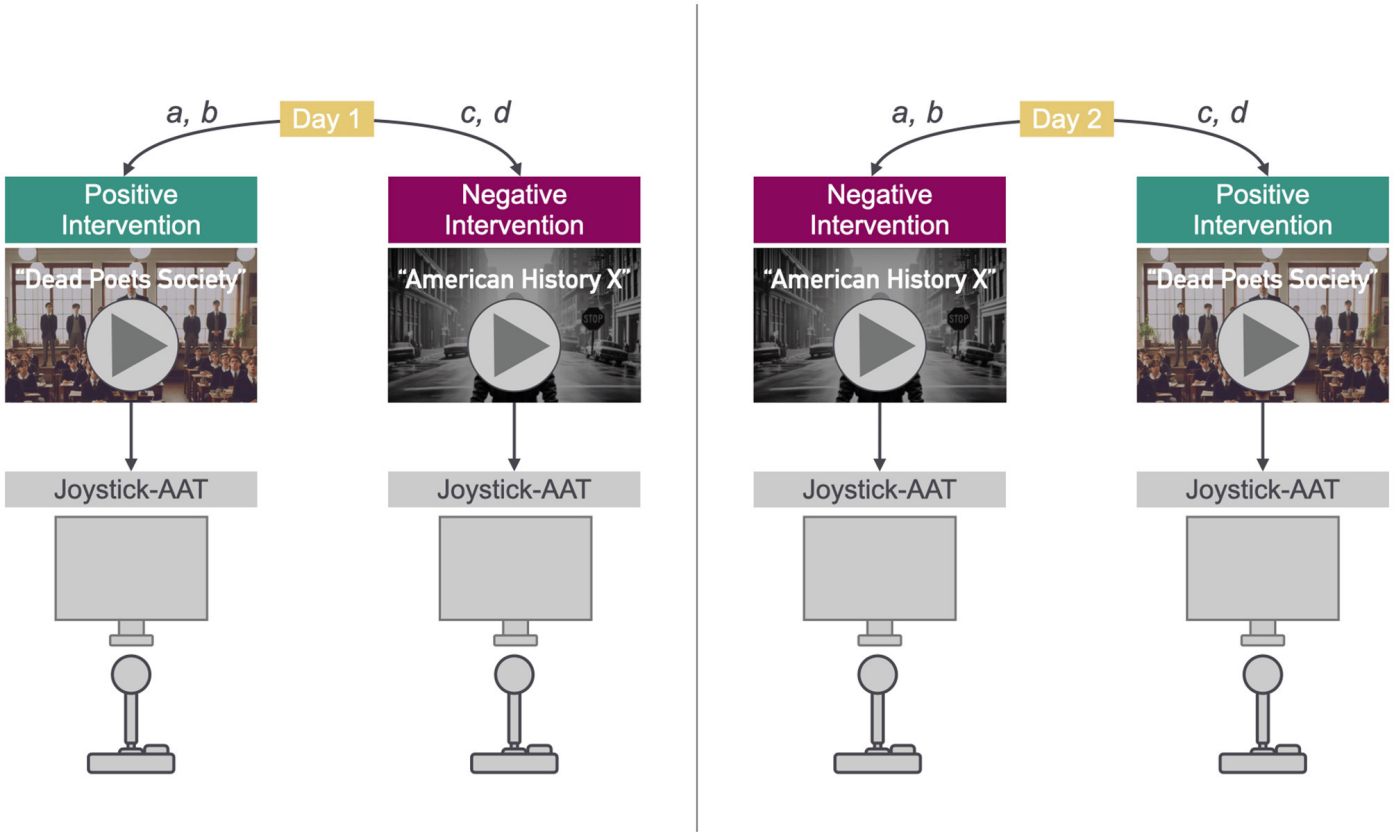
Procedure: Positive/Negative mood induction and AAT. The images used in this figure have been generated with DALL-E 3 (OpenAI, 2023).

The study design is two-factored with two levels each (2 × 2): the intervention aimed at mood induction (*positive, negative*) and AAT instructions (*congruent, incongruent*). Both independent variables have been manipulated within subjects. Figure 1 illustrates the experimental procedure. Reaction time data were collected in a repeated measures design with 88 trials per condition and a total number of 352 trials per subject. The congruent and incongruent AAT instructions were organized in blocks. The order of both positive/negative mood induction and congruent/incongruent AAT block was randomized over participants. We randomly sorted participants into four groups: Groups *a* and *b* underwent positive mood induction in the first session of the experiment and negative mood induction in the second session on a separate day. Groups *c* and *d* watched the negative mood induction video in the first session and watched the positive video in the second session on a separate day. Regarding the AAT block order, participant groups *a* and *c* started with a block with congruent instructions and ended with a block with incongruent instructions. Groups *b* and *d*, on the other hand, started with a block with incongruent instructions and ended with a block with congruent instructions. The participants were equally distributed across groups, 24 participants each belonging to groups a, b and c, and 23 participants belonging to group d. In each experiment session, the participants thus took part in one mood induction condition (positive or negative) and contributed data to both instructions (congruent and incongruent) of the AAT with 44 trials each. The first four trials of each AAT block were practice trials. The two different mood induction conditions were measured in two separate sessions in order to account for any difficulties when trying to induce a positive mood after having induced a negative mood or vice versa. The mean number of days between sessions was 3.56 (*sd* = 7.34, *median* = 2).

At the beginning of the experiment, the participants were asked to provide information on their general medical history, to read the participant information carefully, and to give their written informed consent to participate in the study. Moreover, as the study was conducted during the COVID-19 pandemic, they had to adhere to the 2G status (fully vaccinated or recovered) and to fill in protocols for tracing of contacts. An FFP2 mask had to be worn in the laboratory by both the experimenter and subject during the whole time, and the room was thoroughly and frequently ventilated.

**Figure 2:**
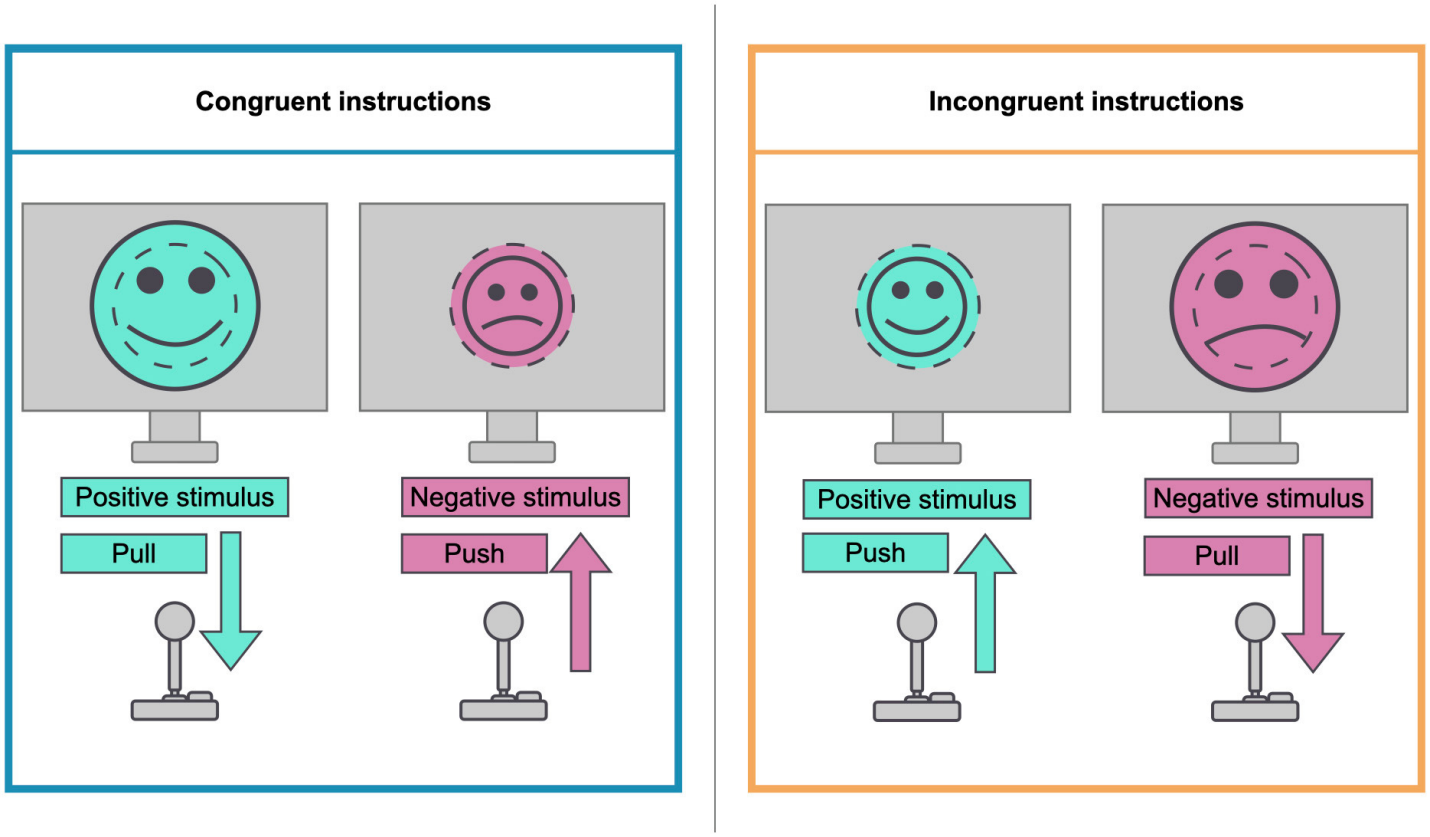
Illustration of congruent and incongruent instructions.

The two sessions of the experiment followed the same general procedure. At the beginning of each of the sessions, the participants completed a 20-item PANAS questionnaire to assess their baseline mood. Ten positive affect (PA) and 10 negative affect (NA) items were rated on a 5-point scale from 1 (very slightly or not at all) to 5 (very much). We chose the PANAS to assess the success of mood induction. This decision was motivated by three main reasons: First, the PANAS has been constructed to measure mood (Watson et al., 1988), and its validity as an affective measurement scale has been repeatedly confirmed (Terraciano et al., 2003; Crawford and Henry, 2004). Second, the PANAS has been used successfully for our specific use case: To check whether a mood induction was successful or not (Rispler et al., 2018; Peel et al., 2023; Rahimi et al., 2019; Palmiero et al., 2015; Guhn et al., 2019). Third, the PANAS provides a clear, simple and fast solution for defining a mood induction success given time constraints during an experiment. After having answered the PANAS, the participants watched a short video according to their mood induction condition (positive or negative). They were instructed to watch the video, listen to its sounds, and try to let their mood be influenced by the video. They were also instructed to try to hold on to that mood during the rest of the experiment. After the intervention, the participants were asked to complete another PANAS questionnaire. Depending on their group, the participants then started the AAT with either a block with congruent (groups *a* and *c*) or incongruent (groups *b* and *d*) instructions. The next three AAT blocks followed with alternating congruent and incongruent instructions. The first four trials of each block were practice trials and were not to be included in the analysis. During each of the blocks, 44 images from the IAPS, half of which served as positive stimuli and the other half as negative stimuli, were presented in random order. Participants were given explicit task instructions requiring them to evaluate the presented stimuli according to their valence. In the congruent condition, they were instructed to react to positive pictures by pulling the joystick toward their body and to react to negative pictures by pushing the joystick away from their body. In the incongruent condition, participants were instructed to react to negative pictures by pulling the joystick toward their body and to react to positive pictures by pushing the joystick away from their body. Figure 2 illustrates the instructions for congruent and incongruent conditions.

The joystick movements were accompanied by a zooming effect. This means that when the participants pulled the joystick, the size of the stimulus presented on the screen increased, and when the participants pushed the joystick, the size of the stimulus decreased. This helps to disambiguate the meaning of the movements by implementing a subject-reference frame (see, e.g., Rinck and Becker, 2007; Seibt et al., 2008; Czeszumski et al., 2021) and has been shown to produce large and replicable effects (Rougier et al., 2018). Furthermore, the participants were instructed to push and pull the joystick to its limits. Between blocks, they had to take a short break of at least 5 s before they could continue with the next block. In general, the participants were able to regulate the onset of the next stimulus by clicking the index finger button on the joystick. This ensured that they were ready for the next trial, and it also meant that the participants were able to take short breaks in between. After completion of the experiment, the participants were debriefed by asking a couple of questions to determine possible problems or difficulties while performing the task. When they participated in the negative intervention, they were offered to watch a short positive film clip in order to counterbalance their possibly ongoing negative mood.

### 2.5 Data analysis procedure

For the data analysis, we adhere to a Bayesian workflow (e.g., Kruschke, 2018; Schad et al., 2020; Nicenboim et al., 2023). For an accessible introduction to Bayesian Statistics in Cognitive Science, we recommend Schad et al. (2020) and Nicenboim et al. (2023). The models we use are hierarchical generalized linear models. One advantage of Bayesian statistics is that the models do not rely on the assumption that the data is normally distributed. Instead, a link function can be specified depending on the data's raw distribution. We use the Bayesian terminology “population-level effects” and “group-level effects” to describe the fixed and random effects known from frequentist statistics, respectively. For reproducibility reasons, we report the following details of the models: we report the number of chains and iterations with which we have run the models and the priors we use. The priors directly influence the posterior distribution, so it is important that they are chosen sensibly and in line with expectations a priori, i.e. before having seen the data. We report a sensitivity analysis of the priors in the Supplementary material. Another advantage of Bayesian statistics is that instead of being confined to binary choices of whether an effect is present or not as in Null-hypothesis-significance-testing (NHST), the focus is more on how much evidence can be gathered in favor or against an effect and on the sizes of the effects. A focus on such alternative analyses that go beyond NHST and the interpretation of a binary 4*p*-value have been argued to increase scientific rigor by the need to think about what the role of each statistic is in interpreting the results (Frias-Navarro et al., 2020). The main effects we report are summarized by a measure of centrality and a measure of the uncertainty about the estimated parameters. As a measure of centrality, we report posterior means, and as a measure of uncertainty, we report 95% Credible Intervals (CrIs) computed on the posterior via the Highest Density Interval (HDI). Aiming for a Bayesian workflow, we do not conduct null hypothesis significance tests and revert from discrete binary decisions regarding our hypotheses and the existence of an effect. Instead, we carefully choose priors based on previous work and compute all statistics on the posterior distribution. The focus of the here conducted analyses is on the magnitude and probability of the estimated parameters expressed by posterior means and CrIs. As an additional basis for understanding the probabilities of the investigated effects, we also report the results of Bayesian tests for the existence and significance of the effects, probability of direction (pd), and Region Of Practical Equivalence (ROPE). The probability of direction defines the part of the posterior distribution that is of the median's sign, i.e. positive if the median is positive and negative if the median is negative. The pd can thus be interpreted as the probability that a parameter's posterior distribution is strictly positive or negative, and it can serve as a test for the existence of an effect (Makowski et al., 2019a). The ROPE defines a “null” region that can be used as a decision criterion for a Bayesian version of null hypothesis significance testing. The null hypothesis is rejected if the HDI lies outside the ROPE completely. It is accepted if the HDI lies entirely inside the ROPE (Kruschke, 2018). For the tests conducted here, we use convenient functions provided by the R package bayestestR (Makowski et al., 2019b). The ROPE range is calculated following Cohen's recommendations, according to which a negligible effect size can be estimated with one-tenth of the standard deviation of the response variable (Cohen, 1988). Some further parameters are important when interpreting the effects and reliability of the models. We include the model outputs as generated by brms in the Supplementary material. There, the main focus is on the effective sample sizes (i.e., do we have enough observations?) and on the Rhat value (i.e., has the model converged?).

All model predictors are treatment-coded. We calculate the relevant contrasts on the posterior distribution. For many of the effects we describe, we compute posterior difference distributions, i.e., posterior distributions that encode a difference between the relevant alternatives. This can be, for example, the difference in reaction times between the congruent and the incongruent condition, or it can be the difference in PA values on the PANAS questionnaire after compared to before the mood induction. We calculate the above-described summary statistics on these posterior difference distributions and interpret them accordingly. All figures show posterior means (presented as large dots) and CrIs computed as HDIs (presented as error bars) on the millisecond timescale, except denoted otherwise.

## 3 Results

### 3.1 Data preprocessing

After the exclusion of the practice trials, the final sample comprises 30,400 complete rows of data from 95 participants, evenly distributed across groups. As preregistered, we excluded response times of trials with incorrect responses and trials with reaction times < 150 ms. 3.48% of the total number of observations (1,059 trials) were excluded for being incorrect. Another 46 trials (0.15%) were excluded due to their reaction times being smaller than 150 ms. We found that the sample also included some unreasonably large response times with values up to 65 s, which drastically exceeds a common range of reaction times. Therefore, we decided to exclude observations with reaction times larger than 2.5 standard deviations from the mean over all measurements, which is a standard procedure for response time analyses (Baayen and Milin, 2010).^4^ This led to an exclusion of 449 trials (1.48%). In total, 5.11% of the collected data (1,554 trials) were excluded due to the above reasons. The final sample analyzed here includes 28,846 observations from 95 participants with reaction times ranging from 166.58 to 2,466.31 ms (*M* = 880.42, *SD* = 305.64).

### 3.2 Preregistered analysis

We have preregistered two hypotheses:

1. Negative mood facilitates performance in the incongruent condition (i.e., *avoiding* positive stimuli and *approaching* negative stimuli).
2. Positive mood facilitates performance in the congruent condition (i.e., *approaching* positive stimuli and *avoiding* negative stimuli).

Based on these hypotheses, we predict that the AAB, i.e., the difference in reaction times between the congruent and incongruent condition, is more pronounced after positive mood induction and less pronounced after negative mood induction. This means that the difference in reaction times between the congruent and incongruent conditions should be smaller after negative mood induction than after positive mood induction. We report descriptive statistics of the reaction time data in the Supplementary material.

To analyze the data, we log-transformed and standardized the data and fit a hierarchical Bayesian model that predicts reaction times in terms of AAT instruction (congruent/incongruent), mood induction (positive/negative), and their interaction, as preregistered. We included group-level effects for participants and trials. In the preregistration, we had planned to run the model with four chains, 2,000 iterations, and a warm-up period of 1,000 iterations. We needed to increase the number of iterations because the effective sample sizes were too low. We thus ran the model with 10,000 iterations and a warm-up period of 2,000 iterations. We preregistered that we wanted to specify slightly skeptical priors, which assume that there is no difference between the two mood induction conditions, and otherwise use priors slightly informed by the data to enhance a better model fit. We performed prior predictive checks to find a set of priors that conform with these goals. We used a normal distribution centered on 0 with a standard deviation of 1 as the intercept prior and a prior agnostic to the direction of an effect [normal (0, 0.05)] as the prior for the population-level effects. As the prior for sigma, we chose normal (0, 0.5); for the standard deviation of group-level effects, we chose normal (0, 0.1); and for the correlation of group-level effects, we chose the LKJ prior [lkj (2)] as suggested by Nicenboim et al. (2023). Prior predictive checks are included in the Supplementary material.

**Figure 3:**
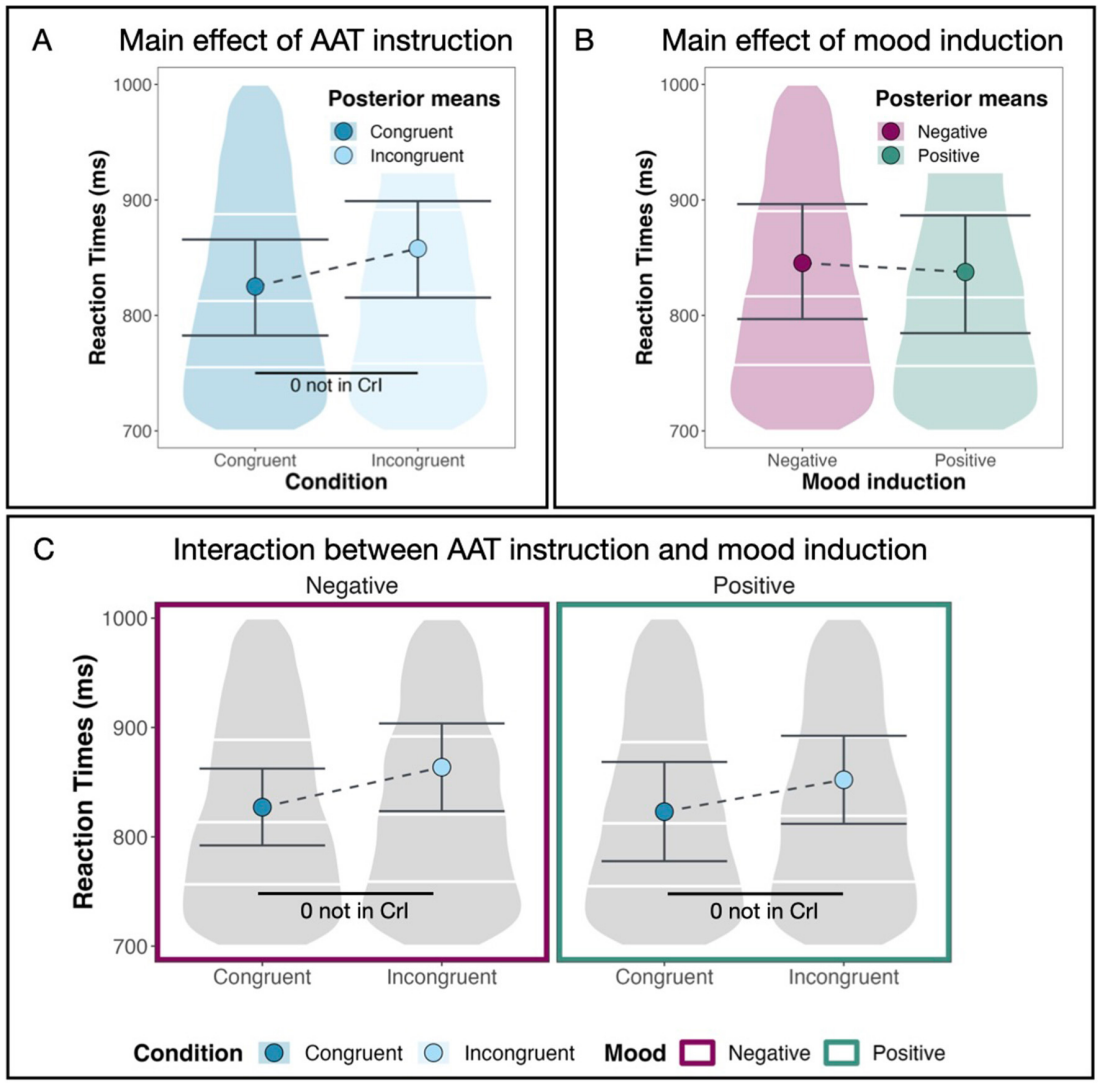
Main effects and two-way interaction. **(A)** The substantial main effect of AAT instruction (congruent vs. incongruent). **(B)** The non-substantial main effect of mood induction (positive vs. negative). **(C)** The non-substantial interaction between AAT instruction and mood induction. Large dots denote posterior means, and the error bars represent 95% CrIs computed as HDIs on the posterior distribution.

We find a main effect for AAT instruction [*M* = 0.11, *CrI* = (0.06, 0.16)] with a 99.99% probability of being positive^5^ and 0.00% of the posterior distribution lying in the ROPE region (see Table 1).^6^ This means that reaction times are larger or, in other words, participants react slower in AAT blocks with incongruent instructions. Figure 3A shows posterior means with 95% CrIs for both congruent and incongruent AAT instructions plotted against the data distribution. We observe faster reaction times in the congruent condition than in the incongruent condition.

**Table 1:** Posterior summary statistics of the preregistered model.

	**Mean**	**l-95% CrI**	**u-95% CrI**	**pd (in %)**	**% in ROPE^*a*^**	**Rhat^*b*^**	**ESS^*c*^**
Intercept	–0.03	–0.14	0.08	71.44	35.51	1.00	1534
AAT instruction (incongruent)	0.11^*^	0.06	0.16	99.99	0.00	1.00	11570
Mood induction (negative)	–0.02	–0.08	0.05	67.49	60.97	1.00	6705
AAT instruction : mood induction	–0.01	–0.06	0.05	60.56	76.60	1.00	17658

^*a*^ROPE range: (–0.03, 0.03), i.e., about 30 ms around 0.

^*b*^Rhat value must be close to 1 (< 1.05) and indicates convergence (Nicenboim et al., 2023).

^*c*^Effective Sample Size (ESS) should be sufficiently large (>1, 000, Bürkner, 2017).

^*^CrI does not include 0.

As shown in Figure 3B, the mood induction did not have a direct influence on the reaction times. Our model estimates the coefficient for negative mood induction at –0.02 with a Credible Interval of (–0.08, 0.05). The probability of it being negative (*pd*) is 67.49%, and 60.97% of the posterior distribution lies in the ROPE region (see Table 1). We conclude that there is no sufficient evidence for a main effect of mood induction on the AAB.

Figure 3C shows that reaction times do not vary much between mood induction conditions. After both positive and negative mood induction, we observe faster reaction times in congruent conditions than in incongruent conditions. We calculate the relevant differences directly on the posterior distributions of the model. The difference in reaction times between the congruent and incongruent conditions is substantial after both positive and negative mood induction with a mean posterior of *M* = 0.10 [*CrI* = (0.04, 0.16), *pd* = 99.91%, 0.00% in *ROPE*] after positive mood induction and of *M* = 0.11 [*CrI* = (0.06, 0.16), *pd* = 99.99%, 0.00% in *ROPE*] after negative mood induction. When we compare how strong the AAB is between mood induction conditions, i.e., how large the difference in reaction times between the congruent and the incongruent condition is in both mood induction conditions, we see no difference between mood induction conditions [*M* = –0.01, *CrI* = (–0.06, 0.05), *pd* = 60.56%, 76.60% in *ROPE*, see Table 2]. This means that we do not find the pattern in the reaction times that we expected according to our hypotheses. In other words, we do not find a facilitating effect for the incongruent condition after negative mood induction and for the congruent condition after positive mood induction. We conclude that the AAB effect does not depend on the type of mood induction but is robust across both mood induction conditions. In other words, mood induction does not have a moderating effect on the AAB.

**Table 2:** Preregistered model: Summary statistics of the posterior difference distributions.

	**Mean**	**l-95% CrI**	**u-95% CrI**	**pd (in%)**	**% in ROPE^*a*^**
Difference between conditions (incongruent – congruent) after positive mood induction	0.10^*^	0.04	0.16	99.91	0.00
Difference between conditions (incongruent – congruent) after negative mood induction	0.11^*^	0.06	0.16	99.99	0.00
Difference in AAB between mood inductions (positive – negative)	–0.01	–0.06	0.05	60.56	76.60

^*a*^ROPE range: (–0.03, 0.03), i.e., about 30 ms around 0.

^*^CrI does not include 0.

### 3.3 Exploratory analyses

#### 3.3.1 PANAS and the success of our mood manipulation

Next, we want to find out whether the reason why we did not find the expected effect of mood induction on the AAB might be due to our mood manipulation. Our goal is to analyze the PANAS questionnaire responses before and after the mood induction and find out whether the reported affect of the participants changed after the mood manipulation compared to before.

**Figure 4:**
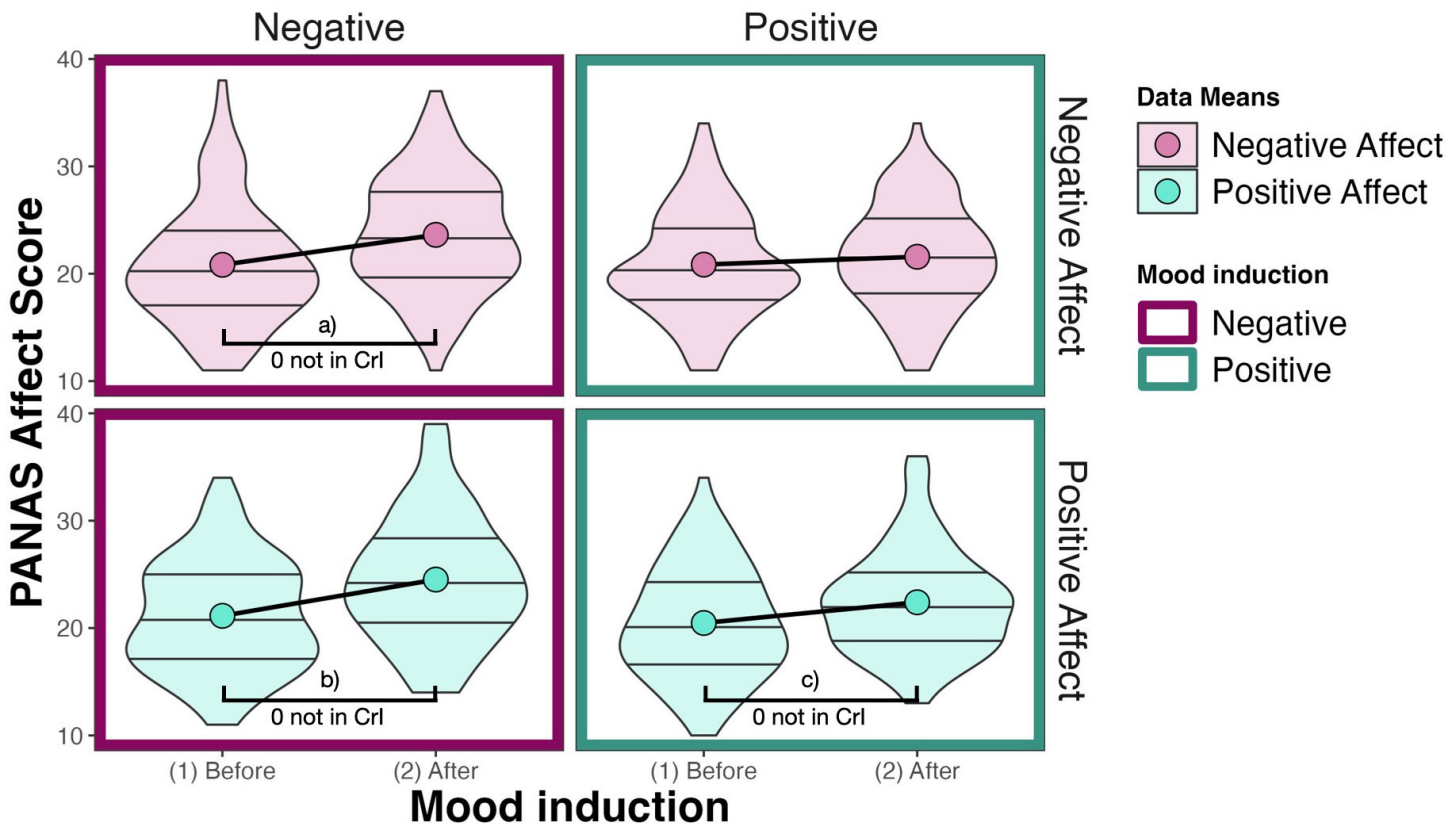
Responses on the PANAS affect scale. Here, large dots denote data means. The PANAS is evaluated for positive and negative affect separately by summing up the values of the responses (1–5) for each item (10 for each affect type). Effects (a–c) are summarized in Table 3.

We expected that a negative mood induction should lead to an increase in NA and that a positive mood induction should lead to an increase in PA. We calculate the PANAS scores by summing up the responses per affect scale as intended by the creators of the scale. The PANAS affect scales (positive and negative) range from 0 to 50. The baseline mood of participants is reflected by a mean of 20.84 (*SD* = 5.2) on the negative affect scale and a mean of 20.81 (*SD* = 5.15) on the positive affect scale. After a positive mood induction, both negative affect (*M* = 21.58, *SD* = 4.91) and positive affect (*M* = 22.4, *SD* = 4.83) increase by less than two points on average. After a negative mood induction, negative affect (*M* = 23.62, *SD* = 5.33) and positive affect (*M* = 24.52, *SD* = 5.84) increase by more than two points on average. The data is visualized in Figure 4. The distribution of the data indicates that while we deal with individual differences, on average, the affective valence of the mood induction does not seem to be reflected in the self-reports of the participants. What we instead see is that both mood manipulations increase the affect scores on average, whereby positive mood induction influences the affect scores less than negative mood induction.

We fit a Bayesian hierarchical model with a Gaussian link function on the PANAS questionnaire data to investigate this further. The model estimates the PANAS affect scores, i.e., summed up values for positive and negative affect scale items separately, by PANAS scale affect (positive/negative), mood induction condition (positive/negative), the questionnaire number, short PrePost (before/after mood induction), and their interaction. We also included by-subject varying intercepts and slopes. The data was treatment coded, and the intercept reflects the positive affect PANAS score before positive mood induction. The model ran for 10,000 iterations with a 2,000-iteration warm-up period. We used regularizing priors to enhance model convergence. The prior on the intercept is a normal distribution centered on 25 (the middle of the PANAS scale) with a wide standard deviation of 10, and the priors for population and group-level effects are normal distributions centered on 0 (and thus agnostic to the direction of the effect) with a standard deviation of 5. We again used the LKJ prior for the correlation between the by-subject intercepts and slopes, as suggested in Nicenboim et al. (2023). The model outputs are included in the Supplementary material. All Rhat values of the fitted model are 1.00, indicating model convergence, and all effective sample sizes are sufficiently high and range between 8,522 and 24,032.^7^

We calculate posterior difference distributions to compare positive and negative affect PANAS scores before and after both mood induction conditions and obtain an estimate of how much evidence there is for an effect. The difference in positive affect after positive mood induction is substantial [*M* = 1.90, *CrI* = (1.09, 2.76), *pd* = 100%, 0% in *ROPE*^8^]. The difference in negative affect score after positive mood induction is not substantial on the other hand [*M* = 0.73, *CrI* = (–0.10, 1.59), *pd* = 95.57%, 31.67% in *ROPE*]. After negative mood induction, the negative affect score increased substantially by 2.77 [*CrI* = (1.64, 3.88), *pd* = 100%, 0% in *ROPE*]. But also the positive affect score increased substantially by 3.37 [*CrI* = (2.52, 4.23) *pd* = 100%, 0% in *ROPE*, see Table 3].

**Table 3:** PANAS model: Summary statistics of the posterior difference distributions.

	**Mean**	**l-95% CrI**	**u-95% CrI**	**pd (in %)**	**in ROPE (in %)^*a*^**
(a) Difference in negative affect after negative mood induction	2.77^*^	1.64	3.88	100.00	0.00
(b) Difference in positive affect after negative mood induction	3.37^*^	2.52	4.23	100.00	0.00
(c) Difference in positive affect after positive mood induction	1.90^*^	1.09	2.76	100.00	0.00
(d) Difference in negative affect after positive mood induction	0.73	–0.10	1.59	95.57	31.67

^*a*^ROPE range: (−0.03, 0.03), i.e., about 30 ms around 0.

^*^CrI does not include 0.

The model results thus confirm the pattern we saw in the data: After both positive and negative mood induction, both positive and negative affect scores increase. Due to both positive and negative scores increasing, it becomes evident that participants do not self-report changes in their affective state that directly relate to the mood induction: After positive mood induction, we would expect only positive affect to increase, and after negative mood induction, we would expect only negative affect to increase. But what we instead see is a general increase in affect scores after both mood induction conditions. We also see that this increase is larger after negative than after positive mood induction.

In summary, while we see substantial differences in the participants' mood after compared to before the mood induction, these differences do not let us conclude that the mood induction worked as expected, i.e., induced a more positive mood after the positive mood induction and a more negative mood after the negative mood induction. Instead of altering the valence of participants' mood, our mood manipulation seems to have increased participants' activation or arousal. This could be a reason why we do not find the expected effect of mood induction on the AAB. However, the data does not let us conclude whether our mood manipulation was unsuccessful or whether the PANAS questionnaire did not capture the participants' change of mood well enough.^9^ This is why we perform additional analyses on the reaction time data to find out whether there are more subtle changes related to our intervention. To draw the readers' attention to the fact that the mood induction likely has not been successful, we choose another terminology for the subsequent analyses and speak of emotional priming instead. The reason for this is that while we do not have evidence for the success of the mood induction in the sense that it induced a specific positive or negative mood, we can definitely say that the participants have watched different video primes prior to participating in the AAT and this might have had observable effects. Therefore, we use the terminology that participants were emotionally primed, rather than that their mood was influenced, for subsequent analyses.

#### 3.3.2 The role of emotional priming and stimulus valence in the AAB

For further exploratory analyses, we have performed model building as recommended by Nicenboim et al. (2023) based on the preregistered model described above. The final model that we report here predicts reaction times with the three-way interaction of AAT instruction (congruent/incongruent), stimulus valence (positive/negative), emotional priming^10^ (positive/negative), and includes group-level effects for individual subjects (by subject varying slopes and intercepts as well as their correlation) and trials (varying slopes and intercepts as well as their correlation). The predictors are treatment-coded. We use the same priors as in the preregistered model described above. The model ran for 20,000 iterations with a warm-up period of 2,000 iterations. All Rhat values of the fitted model are 1.00, indicating model convergence. All effective sample sizes are sufficiently high and range between 2,757 and 51,988.^11^ The model outputs are available in the Supplementary material.

**Figure 5:**
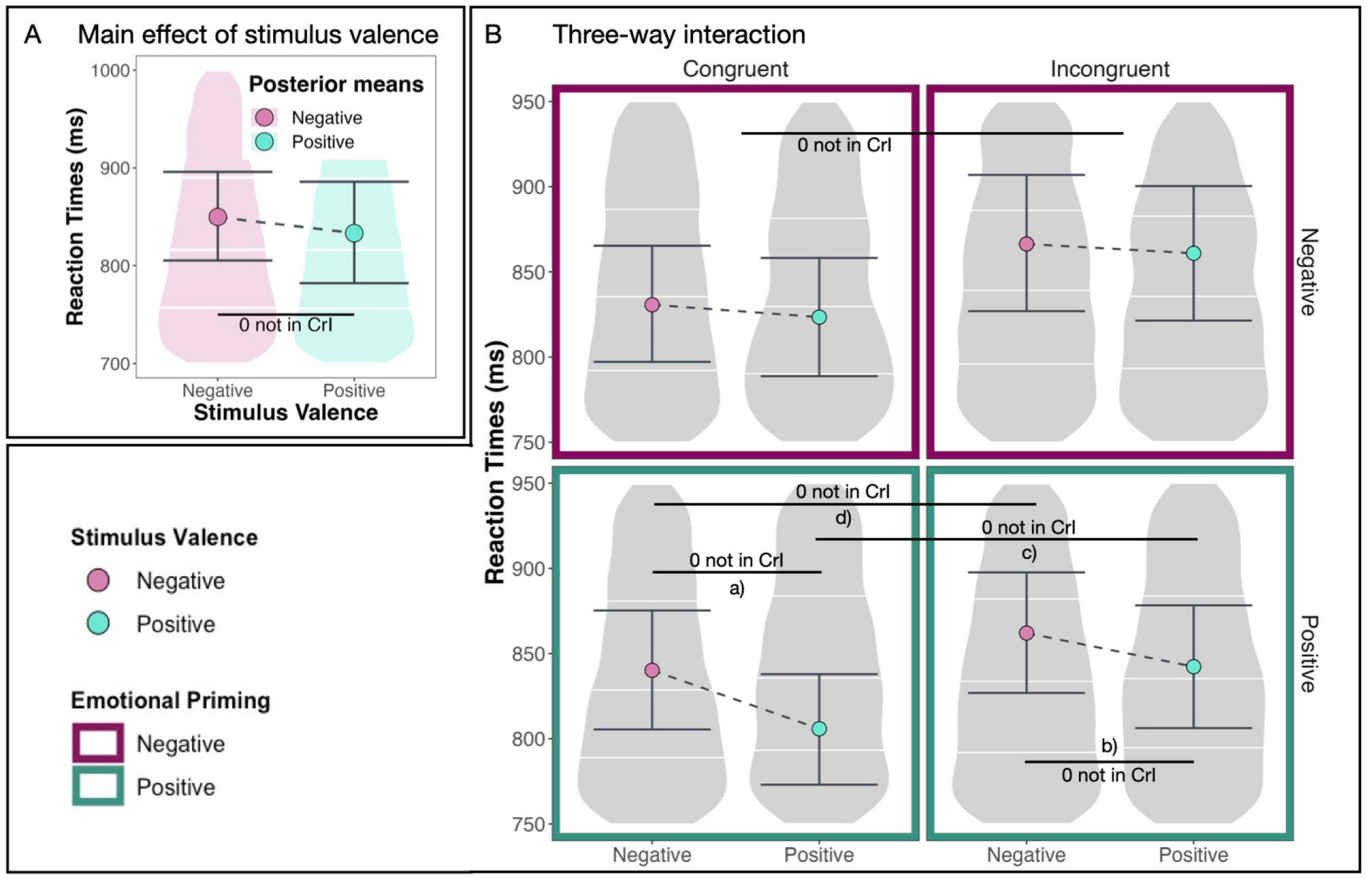
Main effect and interaction. **(A)** The substantial main effect of stimulus valence (positive vs. negative). **(B)** The following three-way interaction: Congruence conditions are displayed on the left (congruent) and on the right (incongruent). Emotional priming conditions are displayed at the top (negative) and the bottom (positive). Stimulus valences are displayed with different colors inside the four panels. Large dots denote posterior means, and the error bars represent 95% CrIs computed as HDIs on the posterior distribution. Effects (a–d) are summarized in Table 4.

First, we make the observation that positive stimuli are reacted to faster than negative stimuli overall (see Figure 5A). This main effect of stimulus valence [*M* = –0.10, *CrI* = (–0.15, –0.06)] is substantial with a 100% probability of being positive and 0.00% of the posterior distribution lying in the ROPE region (see Table 2).

Figure 5B shows our model's predicted reaction times in milliseconds for both congruent and incongruent conditions, both positive and negative emotional priming conditions, and both positive and negative stimuli. In this plot, we observe differences between positive and negative emotional priming. Specifically, we observe that positive stimuli are reacted to especially quickly after positive emotional priming in the congruent condition.

This effect is supported by a substantial difference between reaction times in response to positive and negative stimuli in the congruent condition after positive emotional priming [*M* = –0.10, *CrI* = (–0.15, –0.06), *pd* = 100%, 0% in ROPE, see Table 4a] and by a substantial difference between reaction times in response to positive and negative stimuli in the incongruent condition after positive emotional priming [*M* = –0.07, *CrI* = (–0.12, –0.01), *pd* = 99.33%, 5.48% in ROPE, see Table 4b]. It is further supported by a substantial difference between reaction times in blocks with congruent vs. incongruent AAT instructions after positive emotional priming in response to positive stimuli [*M* = 0.11, *CrI* = (0.04, 0.17), *pd* = 99.87%, 0% in *ROPE*, see Table 4c] and in response to negative stimuli [*M* = 0.08, *CrI* = (0.02, 0.13), *pd* = 99.69%, 2.22% in *ROPE*, see Table 4d]. The differences between reaction times in response to positive vs. negative stimuli differ substantially between emotional priming conditions [*M* = –0.06, *CrI* = (–0.12, –0.01), *pd* = 99.02%, 7.81% in *ROPE*, see Table 4e] with larger differences after positive compared to negative emotional priming. These results are summarized in Table 4.

**Table 4:** Exploratory model: Summary statistics of the posterior difference distributions.

	**Mean**	**l-95% CrI**	**u-95% CrI**	**pd (in %)**	**% in ROPE^*a*^**
a) Main effect of stimulus valence (positive – negative) after positive emotional priming in the congruent condition	–0.10^*^	–0.15	–0.06	100.00	0.00
b) Main effect of stimulus valence (positive – negative) after positive emotional priming in the incongruent condition	–0.07^*^	–0.12	–0.01	99.33	5.48
c) Main effect of AAT instruction (congruent/incongruent): reaction time difference between approaching and avoiding positive stimuli after positive emotional priming^*b*^	0.11^*^	0.04	0.17	99.87	0.00
d) Main effect of AAT instruction (congruent/incongruent): Reaction time difference between avoiding and approaching negative stimuli after positive emotional priming^c^	0.08^*^	0.02	0.13	99.69	2.22
e) Interaction of stimulus valence and emotional priming (positive – negative)^*d*^	–0.06^*^	–0.12	–0.01	99.02	7.81

^*a*^ROPE range: (–0.03, 0.03), i.e., about 30 ms around 0.

^*b*^Computed as the posterior difference between congruence conditions with positive stimuli.

^*c*^Computed as the posterior difference between congruence conditions with positive stimuli.

^*d*^Computed as the posterior difference in stimulus valence between emotional priming conditions.

^*^CrI does not include 0.

To summarize, additional to the previously reported main effects for AAT instruction (congruent/incongruent) and stimulus valence (positive/negative), we find substantial evidence for an interaction between stimulus valence and emotional priming [*M* = –0.06, *CrI* = (–0.12, –0.01), *pd* = 99.02%, 7.81% in *ROPE*]. Specifically, we find that after positive emotional priming, participants react especially fast to positive stimuli. This means that the condition of our AAT after emotional priming, in which participants react the quickest, is when positive stimuli need to be approached in the congruent condition after positive emotional priming. We conclude that the emotional priming had an effect on the AAB. This effect is modulated by stimulus valence.

## 4 Discussion

The goal of the present study was to shed light on the connection between mood and approach-avoidance behavior. Specifically, we were interested in the change of reaction times during an Approach-Avoidance Task after an intervention that aimed at a mood induction. We hypothesized that negative mood would facilitate performance in the incongruent condition and positive mood would facilitate performance in the congruent condition. Thus, we expected a more pronounced AAB, that is a larger difference in reaction times after the positive than after the negative intervention. In our experiment, we found that participants were faster in avoiding negative and approaching positive stimuli (i.e., in the congruent condition) than vice versa (i.e., in the incongruent condition) independent of whether positive or negative videos were shown during the intervention. This provides evidence against our preregistered hypotheses and challenges the idea that negative mood counteracts the AAB. In conclusion, we reproduced the “classic” AAB, i.e. participants were faster in the congruent than in the incongruent condition regardless of the intervention. This could mean that the AAB does not depend on an individual's current mood state. In exploratory analyses, we observed that the mood induction did not cause the intended change on the affective scale of the PANAS. Specifically, while we find substantial differences in responses on the PANAS scales after compared to before the intervention, these differences do not comply with the valence of the intended mood induction in the negative intervention condition: After the positive intervention, only positive affect increased substantially as expected. However, after the negative intervention, both negative and positive affect increased substantially. This could mean that either the intervention did not work as expected or that the PANAS was not suitable to capture the changes in the participants' affective states. Even though the PANAS results did not confirm the expected effects of the intervention, there are measurable behavioral changes in the reaction times after the intervention. This is why we rephrase our intervention as emotional priming. That is, positive/negative priming refers to the condition in which subjects watched videos associated with positive/negative affect, respectively. With this renaming, we connect to literature observing priming effects in the AAB. For example, Smith and Bargh (2008) propose that priming, in their example power priming, influences approach-avoidance tendencies. In our final exploratory analyses, we found that participants were faster in general when reacting to positive than to negative stimuli. Additionally, we found that participants were faster in responding to positive stimuli after positive priming, but we found no such effect for negative priming and stimuli. This suggests that although participants did not self-report a change in mood with respect to the valence of the priming in the negative condition, there still seems to be a significant difference in behavior between negative and positive priming. The following sections aim to interpret the described results within the scientific landscape.

First, concerning the finding of an AAB, i.e., individuals being faster in approaching positive and in avoiding negative stimuli than vice versa (see Figure 3A): The reproduction of the AAB might seem unsurprising at first glance. This very common cognitive bias has been shown many times (e.g., Solzbacher et al., 2022, and see Phaf et al., 2014 for a meta-analysis) and has been hypothesized to represent evolutionary, sensory-motoric (Fridland and Wiers, 2018) or associative-cognitive (e.g., Lavender and Hommel, 2007) tendencies of individuals to be faster in approaching what they need, like or want and to avoid the dangerous or disgusting. However, finding this bias in our specific setup is particularly interesting for several reasons: First, it falsifies one of our hypotheses, namely that being in a negative affective state is counteracting the AAB. We came up with this hypothesis based on results regarding AAB and depression. Specifically, individuals suffering from depression do not exhibit an AAB (e.g. Loijen et al., 2020). We hypothesized that the reason for this could be that those individuals are in a negative emotional state and that this might be the explanation for the difference. However, our findings show that the AAB seems to be robust even after affective priming, also in the explicit-instruction setup [vs. the implicit-instruction setup in Vrijsen et al. (2013)]. Thus, it seems that short-term changes in affective state do not affect an individual's more primal tendencies to interact with encounters in the world. This also emphasizes the severe difference between mere affective state and the mental (and pathological) disorder of depression. While the latter has a strong impact on primal instincts, up to a point where it seems to influence the AAB (e.g., Radke et al., 2014; Becker et al., 2019), the former does not seem to have this impact. Taking an evolutionary perspective on the AAB, this seems reasonable. It would indeed be very unfortunate if having a good or bad day had modulated the reaction of an individual, especially toward negative encounters. On the contrary, it seems to be crucial that primal instincts such as avoiding the dangerous (like running away from a tiger) or disgusting (like keeping one's distance from infectious wounds) stay intact independent of affective states.

Second, we found that after showing positive and negative videos, participants did not report a change in their affective state on the PANAS questionnaire that complies with the valence of the videos used during the intervention (see Figure 4). Instead, they indicated a general increase in activation, with both the negative and positive affect scales showing higher scores after than before the intervention.^12^ This finding can be interpreted in several ways. Firstly, one interpretation is that the priming did not alter the mood of the participants in the expected way. This could be due to various reasons, such as the demographic composition of the participant sample (mostly students) or (un-)suitability of the videos used. Secondly, it is possible that a significant change in affective state occurred, but the PANAS was not suitable to capture it (see, for example, Tuccitto et al., 2010). Given our other findings discussed below, this interpretation seems plausible, but we cannot know for sure whether we induced the intended mood in the participants. There are several reasons why the priming resulted in a behavioral change without altering the PANAS affect scales. First, the PANAS might not be able to capture all kinds of negative and positive change in affective states. The change might have been in a type of affect not captured by the PANAS. For example, the videos might have induced specific forms of negative or positive moods, such as anger or rage, rather than sadness, which the PANAS may not adequately capture. The limitations of the PANAS are discussed in more detail below. Second, participants might not have accurately self-reported their actual affective state due to biases like social desirability bias (“This kind of content does not scare me!”) or an inability to access relevant information. In the end, while the PANAS did not detect a priming-dependent change in mood (and it seems futile to discuss if this is the case), it did detect a priming-independent change. Participants reported higher scores for both positive and negative affective states on the PANAS after both positive and negative priming (see Figure 5), and the change was stronger for negative priming. This could suggest that the participants were affected by the videos, not by changing their affective state but by entering a state of heightened arousal, increasing their general sensitivity to both positive and negative valences.

Third, we found that individuals were generally faster in reacting to positive than to negative stimuli (see Figure 5A). This finding is less straightforward to interpret. Theories explaining reaction time advantages in negative vs. positive stimuli-reactions are widespread. On the one hand, there are evolutionary considerations such as the need to process negative stimuli faster than positive ones (see Irvin et al., 2022 for experimental evidence). On the other hand, several studies have shown faster reaction times to positive than to negative facial expressions (Leppänen et al., 2003) or for processing positive vs. negative information (Unkelbach et al., 2008). The former is explained by the evolutionary need to react to danger as fast as possible, and the latter, for example, by the “density hypothesis.” This hypothesis proposes that there is “a speed advantage in the processing of positive information and […] that this advantage is caused by the higher density of positive information in memory: Positive information is more similar to other positive information, in comparison with the similarity of negative information to other negative information” (Unkelbach et al., 2008, p. 36). While our results are in line with the density hypothesis, the mixed results in the field suggest that there are several aspects influencing the processing and reaction to positive or negative stimuli.

Finally, we found that after positive priming, participants' reaction times were substantially shorter when reacting to positive stimuli (see Figure 5B). The interpretation of these results should be taken with caution due to the aforementioned considerations regarding the success of the intervention and the results of the PANAS questionnaire. It is to say that none of our preregistered hypotheses were confirmed. We did not find an effect of negative priming facilitating incongruent conditions and positive priming facilitating congruent conditions. Based on the observation that subjects suffering from depression do not show an AAB, it has been hypothesized that a healthy population in a negative mood might have similar effects. Our data do not confirm this hypothesis. Thus, the interpretation of the aforementioned effect we observe after positive priming is strictly exploratory. That said, there are a few interpretations that might explain such an effect. First, arguably, reacting to positive pictures in the congruent condition is the only condition that can be described as “pleasant.” Dealing with negative content—independent of the condition—can be described as unpleasant, and seeing something positive but having to avoid it should be less pleasant than approaching the positive content. Thus, it could be argued that the positive priming modified the individual's affective state such that individuals were able to react faster in pleasant conditions compared to unpleasant conditions. Following this argument, our findings could be described as a valence-congruency-match and our manipulation picks up the pleasant/unpleasant dimension rather than the positive/negative one. Following this line of thought, our results suggest that positive priming facilitated pleasant conditions but not unpleasant conditions. An explanation for not finding a similar effect for negative priming could be that positive priming lasts longer than negative priming (e.g., Walker et al., 2003). Thus, it is conceivable that the faster decay of a negative affective state prevented a significant effect from being discovered. Finally, there might just be fundamentally different mechanisms in place for the processing and reaction toward negative and positive stimuli. For example, the before mentioned density hypothesis (Unkelbach et al., 2008) might help explain the difference: Positive information is more similar to other positive information than negative is to other negative information. For example, in negative priming, anger and fear might counteract each other in terms of their influence on approach-avoidance behavior. While anger might facilitate an approach, fear facilitates avoidance behavior (but see Ikeda, 2023b for conflicting evidence). This could be another explanation for why we found an effect for positive priming (where there is no such diversity) and no effect for negative priming.

Ostensibly contradictory to the faster reaction times we found in response to positive stimuli compared to negative stimuli after positive priming, other authors have found seemingly conflicting results. Namely Kaspar et al. (2015), primed participants with positive and negative images and let them explore websites with negatively and positively laden content while tracking the effects of overt attention and exploratory behavior. Unlike us, the authors have shown that participants, after positive priming, were faster in attending to negative stimuli. But similarly to us, the authors only found an effect for positive priming. However, there is an important difference between the two studies: While Kaspar et al. (2015) investigated eye movements, a clear reference to attention, we investigated the reaction to the stimuli. In our study, there is no way of distinguishing the process of processing the stimuli and attending to them from the actual reaction. Instead, our reaction times capture both attention and reaction together. Thus, when comparing our results to the results of Kaspar et al. (2015), the differences in results, might suggest that attention and reaction are indeed very distinct. While positive priming might support attending to negative stimuli over positive ones [study by (Kaspar et al., 2015)], reacting to the respective stimuli seems to be another story (our study). This distinction is also supported by other literature. For example, (Leppänen et al., 2003, p. 113) show that the reaction time advantage for positive stimuli “occurs primarily at premotoric processing stages.” Our findings in relation with these previous findings support the important distinction between attention on the one hand and reaction on the other hand, especially in the context of positive priming.

In the following, we would like to point out some limitations of our study. First, we would like to discuss the stimuli that we used. While driven by our goal to choose objective criteria for our selection of the video stimuli used in the intervention, we ended up using a video in the negative condition that scored highest on NA (2.73), but also scored fairly high on PA (2.04). This might explain why we found an increase in positive affect after the negative intervention in our PANAS results. Additionally, a possible limitation of the video stimuli used in this study is that the stimuli used in the negative and positive intervention are not fully comparable in terms of their duration and color. The video used in the negative condition is shorter than the video used in the positive condition and in black-and-white color instead of full color. This is due to the fact that the videos are from a database that uses movie scenes which are hard to control for. As the stimuli have been rated and shown to elicit a certain mood in an independent rating study by Schaefer et al. (2010), we are confident that our choice of stimuli was justified. However, we cannot rule out that these differences between stimuli impacted our results. Furthermore, the pictures used in the AAT stem from the International Affective Picture System (IAPS) (Lang et al., 1997), which is a well-tested and established database of emotion-inducing affective pictures. Yet, the verification of the IAPS happened almost 30 years ago. Thus, although multiple studies in the field of approach and avoidance have used the database also recently (e.g. Solzbacher et al., 2022; Kaspar et al., 2015) and a recent systematic review (Branco et al., 2023) suggests that the IAPS is still the number one choice for eliciting emotions with pictures, a more recent validation would be desirable.

Second, we discuss the mood induction and our attempts to capture the effect of our intervention with the PANAS. Above, we pointed out already that the results of our intervention—showing positive and negative videos—did not result in the expected effect on the PANAS affect scales. The reasons for why the intervention did not show the expected results have already been discussed above. Here, we would like to discuss the limitations of the PANAS as a tool to assess the success of the intervention in more detail. While the PANAS was used for this purpose in the literature (Rispler et al., 2018; Guhn et al., 2019; Palmiero et al., 2015; Peel et al., 2023; Rahimi et al., 2019), and although the PANAS was specifically designed to capture mood (Watson et al., 1988), it was pointed out by, for example, Ekkekakis and Zenko (2016) that the PANAS might not be fully suitable for this purpose. Specifically, Ekkekakis and Zenko (2016) point out that the PANAS questionnaire only captures high-activation states (e.g., enthusiastic, and not low-activation states such as calmness) and that it mixes different affective states together, i.e. not only mood but also core affect and emotion items. We addressed these limitations in two ways: First, while previous research has focused on a significant change in summed scales after compared to before the mood induction (e.g., Rispler et al., 2018; Peel et al., 2023), we performed a more fine-grained analysis of the PANAS data, taking into account the valence of the scale items. Second, we performed a Principal Components Analysis (PCA) of the scale items to identify possible sub-scales that might be used for a more fine-grained analysis differentiating between, for example, items that indicate high- and low-activation states. The results of the PCA can be found in the Supplementary material but they have not led to the identification of subscales. This might mean that the items are so disentangled that they should not be interpreted individually but rather as summed scales for positive and negative affect, respectively, as instructed by the authors of the PANAS (Watson et al., 1988). For future research, we propose to use scale-sensitive analyses of the PANAS to interpret changes in mood. A future research endeavor might also be to construct an alternative assessment procedure to the PANAS that, in addition to the valence scale (positive vs. negative), also involves an activation or arousal scale (high-activation vs. low-activation). This would allow to disentangle the dimensions valence and arousal better in future research.

Finally, we would like to discuss some possible limitations that came up during the review process. First, we do not have a neutral mood induction condition but only compare a positive with a negative intervention. This choice of study design is justified by two main reasons: Firstly, neutral affect might not be the best baseline because (a) neutral affect has been argued to be an affective state that can co-occur but does not correlate with positive or negative affect that is worth investigating on its own (Gasper et al., 2019) and (b) it is difficult to disentangle whether a neutral condition really induced a neutral mood or just preserved the mood state a participant came with to the lab or even induced a mood that more resembles positive mood (see e.g. Devilly and O'Donohue, 2021 for a discussion on this matter). Secondly, from a methodological point of view, adding another level to the factor (i.e. a neutral condition) comes with the need of collecting more data to get the same statistical power. Adding another level to an experiment design can let the required sample size increase exponentially. As adding this level was not strictly necessary to assess the difference we are interested in, i.e. positive vs. negative, we decided against a neutral condition in line with a resource management perspective (Collins et al., 2009). However, we would like to point out that this means that our interpretations are confined to this setup, i.e. we cannot make conclusions about a difference between positive and neutral affect but only about the difference between positive and negative affect. Second, our experiment was not designed to study the effects of mood induction and stimulus valence in isolation. Since both primes and stimuli are inherently charged with positive and negative valence, an interaction of these factors cannot be ruled out. However, for our research question, this seems unavoidable. In our setup, our goal was not to disentangle these factors but rather to study them together and then use statistical models to gain insights into their relationships and interactions. Third, apart from the two PANAS questionnaires and the reaction times, we have no additional insight into the subjects' wellbeing, affective state, or psychological state. Thus, other factors concerning the psychological state of the subjects might have influenced the results. As is best practice, we included group-level effects over both trials and participants to account for unknown or unmeasurable parameters within groups. Fourth, valence involves more nuance than simply positive or negative. As already pointed out earlier in the discussion section, the traditional assumption that positive emotions drive approach behaviors while negative emotions drive avoidance behaviors can be questioned. Specifically, there are negative affective states like anger that are associated with (spatial) approach and positive affective states like resentment that can be associated with (spatial) avoidance. The negative prime we used was a video that could very well be characterized as inducing anger rather than other negative emotions such as sadness or fear, which could have affected the outcome of our experiment. Recent research suggests that the specific category of the emotionally valenced stimuli might matter more in avoidance than in approach (van Alebeek et al., 2023). Future research could provide more insight into the question how different types of negative affect influence the AAB, for example by using mood induction procedures that induce a different negative affective state than the one targeted in our mood induction, such as sadness or fear. Lastly, while there is literature on the duration and sustainability of mood vs. emotion, it has been suggested that there is some transcendence between different affective states such as mood and emotion. Thus, it cannot be conclusively stated that our intervention affected (only) mood and no other affective states. Rather, an impervious separation of different affective states seems artificial in this context.

Despite being studied a lot, attempts to explain the mechanisms of approach-avoidance behavior are still not exhaustive and a lot of questions remain open. Future studies should be conducted to add to the evidence concerning the role of affect in the AAB. For example, researchers might want to experiment with the specific type of mood induction. One idea would be to induce sad mood in the negative intervention condition. This might be a good way to more specifically test depression-related approach-avoidance motivations. Another idea brought up by one of our reviewers is to use a mood induction with music and let the music play in the background while participants do the AAT. Recently, the test-retest reliability of the AAB has been questioned (Zech et al., 2023). While there is a vast amount of studies finding an AAB, there have been also some failed replication attempts (see e.g. Wiers et al., 2017, for smoking, Galvin et al., 2023, for gambling). One reason for such failed replication attempts and low test-retest reliability might be the difference in mood of the participants taking part in such studies. Usually, such differences are not controlled for. The recent development of smartphone-based AATs (Zech et al., 2023) opens up the possibility to conduct a large-scale study where participants first rate their mood with a psychological scale like the PANAS and then do the AAT. Also with regards to the AAT, there are a number of ideas for future research: Apart from the promising smart-phone based applications mentioned before, one idea will be to move beyond approach-avoidance reactions with a joystick, i.e. arm movement toward full body movements either in Virtual Reality (e.g. Eder et al., 2021; Eiler et al., 2019) or in real life. For example, postural measurement has been applied successfully to investigate approach-avoidance behavior in response to imagined painful vs. non-painful situations (Lelard et al., 2013) and this method might open up future research possibilities on the embodied nature of the AAB. Future research should also be conducted on the level of neural mechanisms and not just behaviorally as is done here. For example, it has been shown that specific neuronal characteristics can lead to maladaptive approach-avoidance behavior in the field of anxiety-related avoidance behavior (Laricchiuta et al., 2023; Aupperle and Martin, 2010; La-Vu et al., 2020). Specifically, Laricchiuta et al. (2023) found in research with mice that individuals with inefficient fear extinction may show increased avoidance tendencies when faced with potential threats, which impacts their decision-making processes. This can have implications for understanding avoidance tendencies in humans and for designing treatments for approach-avoidance related disorders such as anxiety and depression. Future research should focus more on the synergies between neural-level and behavioral research findings to allow for a holistic understanding of approach and avoidance motivation (see e.g. Sütçübaşı et al., 2024 for a recent study combining behavioral and neuroimaging methods). After there has been some recent evidence that the action as such (i.e., the part of the body that the reaction toward a stimulus is performed with) might play an important role for the AAB (Eder et al., 2021; Fridland and Wiers, 2018; Solzbacher et al., 2022), it seems convincing to put a focus on the investigation of the differences between mere attention and premotoric processing stages on the one hand and bodily reactions targeting the stimuli on the other hand in future research. A reflection of the results of our study within the scientific field underlined that different mechanisms might be at play for attending and reacting to valenced stimuli, especially after positive priming. Further studies are needed to investigate these differences and disentangle the mechanisms of different stages in premotoric processing and action, and additional studies might focus on why those mechanisms are only found after positive priming but not after negative priming. Evolutionary ideas have put the focus of science on the importance of negative emotions for a long time, but sometimes it is the positive that has the bigger effect on us.

## Data Availability

The datasets presented in this study can be found in online repositories. The names of the repository/repositories and accession number(s) can be found below: OSF repository: https://osf.io/uvfh9/. The analysis scripts and models are also available at https://osf.io/84th6/.

## Footnotes

^1^There are also other tasks that are used to investigate approach-avoidance behavior, e.g. the manikin task (van Dessel et al., 2018). However, these are not discussed in detail here because they are not the focus of the current study.

^2^Note that unfortunately, there is no meta-analysis yet on this subject as to our knowledge.

^3^Note that the two videos used for the different conditions differ in length and color. The potential impact of these differences is discussed in the limitations section in the discussion.

^4^This exclusion was not preregistered but justified by domain knowledge and common procedures.

^5^For reference for users of the frequentist statistics paradigm: This corresponds to a *p*-value of < 0.001.

^6^The ROPE region of (0.03, –0.03) was calculated by multiplying 0.1 with the standard deviation of the raw reaction times.

^7^Bürkner (2017) recommends ESS > 1, 000 for stable estimates in most models.

^8^The ROPE region was calculated according to recommendations by Kruschke (2018) by using one-tenth of the standard deviation of the response variable around the null. According to Cohen (1988), this results in a negligible effect size.

^9^The PANAS questionnaire includes items of high-activation. This might lead to biases in the measurement of affect. High-activation states might be captured well in contrast to low-activation states.

^10^In previous sections, this variable was called “mood induction.”

^11^Bürkner (2017) recommends ESS > 1, 000 for stable estimates in most models.

^12^Specifically, we observe a substantial increase of only positive affect after positive intervention and a substantial increase of both positive and negative affect after negative intervention.

## References

[B1] Aupperle R. L. Martin P. P. (2010). Neural systems underlying approach and avoidance in anxiety disorders. Dialogues Clin. Neurosci. 12:517. 10.31887/DCNS.2010.12.4/raupperle 21319496 PMC3181993

[B2] Baayen R. H. Milin P. (2010). Analyzing reaction times. Int. J. Psychol. Res. 3, 12–28. 10.21500/20112084.807

[B3] Becker E. S. Barth A. Smits J. A. J. Beisel S. Lindenmeyer J. Rinck M.. (2019). Positivity-approach training for depressive symptoms: a randomized controlled trial. J. Affect. Disord. 245, 297–304. 10.1016/j.jad.2018.11.042 30439675

[B4] Beedie C. Terry P. Lane A. (2005). Distinctions between emotion and mood. Cogn. Emot. 19, 847–878. 10.1080/02699930541000057

[B5] Beevers C. G. Clasen P. C. Enock P. M. Schnyer D. M. (2015). Attention bias modification for major depressive disorder: effects on attention bias, resting state connectivity, and symptom change. J. Abnorm. Psychol. 124, 463–475. 10.1037/abn0000049 25894440 PMC4573770

[B6] Booth C. Spronk D. Grol M. Fox E. (2018). Uncontrolled eating in adolescents: the role of impulsivity and automatic approach bias for food. Appetite 120, 636–643. 10.1016/j.appet.2017.10.024 29066344 PMC5689136

[B7] Bouman D. Stins J. F. Beek P. J. (2022). Both distance change and movement goal affect whole-body approach-avoidance behavior. Psychol. Neurosci. 14, 156–172. 10.1037/pne000025535588387

[B8] Branco D. Gonçalves O. F. Badia S. B. I. (2023). A systematic review of international affective picture system (IAPS) around the world. Sensors 23:3866. 10.3390/s23083866 37112214 PMC10143386

[B9] Bürkner P.-C. (2017). brms: an R package for Bayesian multilevel models using Stan. J. Stat. Softw. 80, 1–28. 10.18637/jss.v080.i01

[B10] Chen M. Bargh J. A. (1999). Consequences of automatic evaluation: immediate behavioral predispositions to approach or avoid the stimulus. Pers. Soc. Psychol. Bull. 25, 215–224. 10.1177/0146167299025002007

[B11] Cohen J. (1988). Statistical Power Analysis for the Behavioral Sciences, 2nd Edn. New York, NY: Routledge.

[B12] Collins L. M. Dziak J. J. Li R. (2009). Design of experiments with multiple independent variables: a resource management perspective on complete and reduced factorial designs. Psychol. Methods 14:202. 10.1037/a0015826 19719358 PMC2796056

[B13] Core Team R. (2022). R: A Language and Environment for Statistical Computing. Vienna: R Foundation for Statistical Computing.

[B14] Cousijn J. Goudriaan A. E. Wiers R. W. (2011). Reaching out towards cannabis: approach-bias in heavy cannabis users predicts changes in cannabis use. Addiction 106, 1667–1674. 10.1111/j.1360-0443.2011.03475.x 21518067 PMC3178782

[B15] Crawford J. R. Henry J. D. (2004). The positive and negative affect schedule (PANAS): construct validity, measurement properties and normative data in a large non-clinical sample. Br. J. Clin. Psychol. 43, 245–265. 10.1348/0144665031752934 15333231

[B16] Czeszumski A. Albers F. Walter S. König P. (2021). Let me make you happy, and i'll tell you how you look around: using an approach-avoidance task as an embodied emotion prime in a free-viewing task. Front. Psychol. 12:604393. 10.3389/fpsyg.2021.604393 33790829 PMC8005526

[B17] Devilly G. J. O'Donohue R. P. (2021). A video is worth a thousand thoughts: comparing a video mood induction procedure to an autobiographical recall technique. Aust. J. Psychol. 73, 438–451. 10.1080/00049530.2021.1997553

[B18] Dickson H. Kavanagh D. J. MacLeod C. (2016). The pulling power of chocolate: effects of approach-avoidance training on approach bias and consumption. Appetite 99, 46–51. 10.1016/j.appet.2015.12.026 26725150

[B19] Eder A. B. Krishna A. Sebald A. Kunde W. (2021). Embodiment of approach-avoidance behavior: motivational priming of whole-body movements in a virtual world. Motiv. Sci. 7, 133–144. 10.31219/osf.io/2rftv

[B20] Eiler T. J. Grunewald A. Brück R. (2019). “Fighting substance dependency combining AAT therapy and Virtual Reality with game design elements,” in Proceedings of the 14th International Joint Conference on Computer Vision, Imaging and Computer Graphics Theory and Applications (VISIGRAPP 2019) (Prague: SciTePress), 28–37. 10.5220/0007362100280037

[B21] Ekkekakis P. Zenko Z. (2016). “Measurement of affective responses to exercise: from “affectless arousal” to “the most well-characterized” relationship between the body and affect,” in Emotion Measurement, ed. H. L. Meiselman (Cambridge: Woodhead Publishing), 299–321. 10.1016/B978-0-08-100508-8.00012-6

[B22] Ernst L. H. Plichta M. M. Dresler T. Zesewitz A. K. Tupak S. V. Haeussinger F. B.. (2014). Prefrontal correlates of approach preferences for alcohol stimuli in alcohol dependence. Addict. Biolol. 19, 497–508. 10.1111/adb.12005 23145772

[B23] Fernández-Aguilar L. Navarro-Bravo B. Ricarte J. Ros L. Latorre J. M. (2019). How effective are films in inducing positive and negative emotional states? A meta-analysis. PLoS ONE 14:e0225040. 10.1371/journal.pone.0225040 31751361 PMC6872151

[B24] Frias-Navarro D. Pascual-Llobell J. Pascual-Soler M. Perezgonzalez J. Berrios-Riquelme J. (2020). Replication crisis or an opportunity to improve scientific production? Eur. J. Educ. 55, 618–631. 10.1111/ejed.12417

[B25] Fridland E. Wiers C. E. (2018). Addiction and embodiment. Phenomenol. Cogn. Sci. 17, 15–42. 10.1007/s11097-017-9508-0

[B26] Fridland E. Wiers C. E. Rinck M. Becker E. S. Gladwin T. E. (2022). An experimental test of integrating imagery with approach bias modification for alcohol: a cautionary tale. Br. J. Health Psychol. 28, 383–396. 10.1111/bjhp.12630 36336992

[B27] Frijda N. H. (1994). Varieties of Affect: Emotions and Episodes, Moods, and Sentiments. New York, NY: Oxford University Press, 59–67.

[B28] Galvin H. R. Boffo M. Snippe L. Collins P. Pronk T. Salemink E.. (2023). Losing sight of Luck: automatic approach tendencies toward gambling cues in Canadian moderate- to high-risk gamblers – a replication study. Addict. Behav. 145:107778. 10.1016/j.addbeh.2023.107778 37364524

[B29] Gasper K. Spencer L. A. Hu D. (2019). Does neutral affect exist? How challenging three beliefs about neutral affect can advance affective research. Front. Psychol. 10:2476. 10.3389/fpsyg.2019.02476 31787911 PMC6856204

[B30] Gerrards-Hesse A. Spies K. Hesse F. W. (1994). Experimental inductions of emotional states and their effectiveness: a review. Br. J. Psychol. 85, 55–78. 10.1111/j.2044-8295.1994.tb02508.x

[B31] Gladwin T. E. ter Mors–Schulte M. J. H. Ridderinkhof K. R. Wiers R. W. (2013). Medial parietal cortex activation related to attention control involving alcohol cues. Front. Psychiatry 4:174. 10.3389/fpsyt.2013.00174 24391604 PMC3868991

[B32] Gomez P. Zimmermann P. G. Guttormsen Schär S. Danuser B. (2009). Valence lasts longer than arousal. J. Psychophysiol. 23, 7–17. 10.1027/0269-8803.23.1.7

[B33] Grèzes J. Erblang M. Vilarem E. Quiquempoix M. Van Beers P. Guillard M.. (2021). Impact of total sleep deprivation and related mood changes on approach-avoidance decisions to threat-related facial displays. Sleep 44:zsab186. 10.1093/sleep/zsab186 34313789 PMC8664577

[B34] Guhn A. Steinacher B. Merkl A. Sterzer P. Köhler S. (2019). Negative mood induction: affective reactivity in recurrent, but not persistent depression. PLoS ONE 14:e0208616. 10.1371/journal.pone.0208616 30645583 PMC6333350

[B35] Heuer K. Rinck M. Becker E. S. (2007). Avoidance of emotional facial expressions in social anxiety: the approach-avoidance task. Behav. Res. Ther. 45, 2990–3001. 10.1016/j.brat.2007.08.010 17889827

[B36] Hommel B. Müsseler J. Aschersleben G. Prinz W. (2001). The theory of event coding (TEC): a framework for perception and action planning. Behav. Brain Sci. 24, 849–878; discussion 878–937. 10.1017/S0140525X01000103 12239891

[B37] Ikeda S. (2023a). Approach-avoidance behavioural patterns towards an affective voice. Int. J. Psychol. 58, 164–172. 10.1002/ijop.12886 36585807

[B38] Ikeda S. (2023b). Examining approach–avoidance responses to facial expressions using a tablet device. Curr. Psychol. 42, 14171–14174. 10.1007/s12144-022-02767-y

[B39] Irvin R. L. Klein R. J. Robinson M. D. (2022). Faster, stronger, and more obligatory? A temporal analysis of negative (versus positive) emotional reactions. J. Exp. Soc. Psychol. 99:104272. 10.1016/j.jesp.2021.104272

[B40] Izard C. E. Dougherty F. E. Bloxom B. M. Kotsch N. E. (1974). The Differential Emotions Scale: A Method of Measuring the Meaning of Subjective Experience of Discrete Emotions. Nashville: Vanderbilt University, Department of Psychology.

[B41] Kaspar K. Gameiro R. R. König P. (2015). Feeling good, searching the bad: positive priming increases attention and memory for negative stimuli on webpages. Comput. Human Behav. 53, 332–343. 10.1016/j.chb.2015.07.020

[B42] Kaye T. (Director). (1998). American History X [Film]. Burbank, CA: New Line Cinema; Savoy Pictures; The Turman-Morrissey Company.

[B43] Kleiner M. Brainard D. Pelli D. (2007). What's new in psychtoolbox-3? Perception 36, 1–16. 10.1177/03010066070360S101

[B44] Krehbiel J. Halbeisen G. Kühn S. Erim Y. Paslakis G. (2021). Too hot to handle: mood states moderate implicit approach vs. avoidance tendencies toward food cues in patients with obesity and active binge eating disorder. J. Psychiatr. Res. 143, 302–308. 10.1016/j.jpsychires.2021.09.031 34530341

[B45] Krieglmeyer R. Deutsch R. (2010). Comparing measures of approach-avoidance behaviour: the manikin task vs. two versions of the joystick task. Cogn. Emot. 24, 810–828. 10.1080/02699930903047298

[B46] Krieglmeyer R. Deutsch R. de Houwer J. de Raedt R. (2010). Being moved: valence activates approach-avoidance behavior independently of evaluation and approach-avoidance intentions. Psychol. Sci. 21, 607–613. 10.1177/0956797610365131 20424109

[B47] Kruschke J. K. (2018). Rejecting or accepting parameter values in Bayesian estimation. Adv. Methods Pract. Psychol. Sci. 1, 270–280. 10.1177/2515245918771304

[B48] Lang P. J. Bradley M. M. Cuthbert B. N. (1997). International Affective Picture System (IAPS): Technical Manual and Affective Ratings. Bethesda, MD: NIMH Center for the Study of Emotion and Attention.

[B49] Laricchiuta D. Gimenez J. Sciamanna G. Termine A. Fabrizio C. Della Valle F.. (2023). Synaptic and transcriptomic features of cortical and amygdala pyramidal neurons predict inefficient fear extinction. Cell Rep. 42:113066. 10.1016/j.celrep.2023.113066 37656620

[B50] Lavender T. Hommel B. (2007). Affect and action: towards an event-coding account. Cogn. Emot. 21, 1270–1296. 10.1080/02699930701438152

[B51] La-Vu M. Tobias B. C. Schuette P. J. Adhikari A. (2020). To approach or avoid: an introductory overview of the study of anxiety using rodent assays. Front. Behav. Neurosci. 14:145. 10.3389/fnbeh.2020.00145 33005134 PMC7479238

[B52] Lelard T. Montalan B. Morel M. F. Krystkowiak P. Ahmaidi S. Godefroy O.. (2013). Postural correlates with painful situations. Front. Hum. Neurosci. 7:4. 10.3389/fnhum.2013.00004 23386816 PMC3564009

[B53] Leppänen J. M. Tenhunen M. Hietanen J. K. (2003). Faster choice-reaction times to positive than to negative facial expressions: the role of cognitive and motor processes. J. Psychophysiol. 17, 113–123. 10.1027//0269-8803.17.3.113

[B54] Loijen A. Vrijsen J. N. Egger J. I. M. Becker E. S. Rinck M. (2020). Biased approach-avoidance tendencies in psychopathology: a systematic review of their assessment and modification. Clin. Psychol. Rev. 77:101825. 10.1016/j.cpr.2020.101825 32143108

[B55] Luo X. Ikani N. Barth A. Rengers L. Becker E. Rinck M.. (2015). Attention bias modification in specific fears: spiders versus snakes. J. Behav. Ther. Exp. Psychiatry 49(Pt A), 30–36. 10.1016/j.jbtep.2015.04.006 25958822

[B56] Makowski D. Ben-Shachar M. S. Chen S. H. A. Lüdecke D. (2019a). Indices of effect existence and significance in the bayesian framework. Front. Psychol. 10:2767. 10.3389/fpsyg.2019.02767 31920819 PMC6914840

[B57] Makowski D. Ben-Shachar M. S. Lüdecke D. (2019b). bayestestr: describing effects and their uncertainty, existence and significance within the bayesian framework. J. Open Source Softw. 4:1541. 10.21105/joss.0154119323591

[B58] Neumann R. Strack F. (2000). Approach and avoidance: the influence of proprioceptive and exteroceptive cues on encoding of affective information. J. Pers. Soc. Psychol. 79, 39–48. 10.1037/0022-3514.79.1.39 10909876

[B59] Nicenboim B. Schad D. Vasishth S. (2023). An Introduction to Bayesian Data Analysis for Cognitive Science. Available at: https://vasishth.github.io/bayescogsci/book/ (accessed May 31, 2023).

[B60] OpenAI (2023). DALL-E 3. OpenAI. Available at: https://openai.com/dall-e-3

[B61] Palmiero M. Nori R. Rogolino C. D'Amico S. Piccardi L. (2015). Situated navigational working memory: the role of positive mood. Cogn. Process. 16, 327–330. 10.1007/s10339-015-0670-4 26216759

[B62] Peel N. Nguyen K. Tannous C. (2023). The impact of campus-based therapy dogs on the mood and affect of university students. Int. J. Environ. Res. Public Health 20:4759. 10.3390/ijerph20064759 36981667 PMC10048764

[B63] Peeters M. Wiers R. W. Monshouwer K. van de Schoot R. Janssen T. Vollebergh W. A. M.. (2012). Automatic processes in at-risk adolescents: the role of alcohol-approach tendencies and response inhibition in drinking behavior: alcohol use in at-risk adolescents. Addiction 107, 1939–1946. 10.1111/j.1360-0443.2012.03948.x 22632107

[B64] Phaf R. H. Mohr S. E. Rotteveel M. Wicherts J. M. (2014). Approach, avoidance, and affect: a meta-analysis of approach-avoidance tendencies in manual reaction time tasks. Front. Psychol. 5:378. 10.3389/fpsyg.2014.00378 24847292 PMC4021119

[B65] Radke S. Güths F. André J. A. Müller B. W. de Bruijn E. R. A. (2014). In action or inaction? Social approach-avoidance tendencies in major depression. Psychiatry Res. 219, 513–517. 10.1016/j.psychres.2014.07.011 25060832

[B66] Rahimi M. Sabahi P. Bigdeli I. (2019). The effect of induced positive and negative mood on creativity. Int. J. Psychol. 13, 5–21. 10.24200/ijpb.2018.115424.

[B67] Rinck M. Becker E. S. (2007). Approach and avoidance in fear of spiders. J. Behav. Ther. Exp. Psychiatry 38, 105–120. 10.1016/j.jbtep.2006.10.001 17126289

[B68] Rispler C. Luria G. Kahana A. Rosenblum S. (2018). Mood impact on automaticity of performance: handwriting as exemplar. Cognit. Comput. 10, 398–407. 10.1007/s12559-017-9540-y

[B69] Rotteveel M. Phaf R. H. (2004). Automatic affective evaluation does not automatically predispose for arm flexion and extension. Emotion 4, 156–172. 10.1037/1528-3542.4.2.156 15222853

[B70] Rougier M. Muller D. Ric F. Alexopoulos T. Batailler C. Smeding A.. (2018). A new look at sensorimotor aspects in approach/avoidance tendencies: the role of visual whole-body movement information. J. Exp. Soc. Psychol. 76, 42–53. 10.1016/j.jesp.2017.12.004

[B71] Schad D. Betancourt M. Vasishth S. (2020). Toward a principled bayesian workflow in cognitive science. Psychol. Methods 26, 103–126. 10.1037/met0000275 32551748

[B72] Schaefer A. Nils F. Sanchez X. Philippot P. (2010). Assessing the effectiveness of a large database of emotion-eliciting films: a new tool for emotion researchers. Cogn. Emot. 24, 1153–1172. 10.1080/02699930903274322

[B73] Seibt B. Neumann R. Nussinson R. Strack F. (2008). Movement direction or change in distance? Self- and object-related approach–avoidance motions. J. Exp. Soc. Psychol. 44, 713–720. 10.1016/j.jesp.2007.04.013

[B74] Smith P. K. Bargh J. A. (2008). Nonconscious effects of power on basic approach and avoidance tendencies. Soc. Cogn. 26, 1–24. 10.1521/soco.2008.26.1.1 18568085 PMC2435045

[B75] Solarz A. K. (1960). Latency of instrumental responses as a function of compatibility with the meaning of eliciting verbal signs. J. Exp. Psychol. 59, 239–245. 10.1037/h0047274 13832584

[B76] Solzbacher J. Czeszumski A. Walter S. König P. (2022). Evidence for the embodiment of the automatic approach bias. Front. Psychol. 13:797122. 10.3389/fpsyg.2022.797122 36160565 PMC9505509

[B77] Sütçübaşı B. Bayram A. Metin B. Demiralp T. (2024). Neural correlates of approach–avoidance behavior in healthy subjects: effects of low-frequency repetitive transcranial magnetic stimulation (rTMS) over the right dorsolateral prefrontal cortex. Int. J. Psychophysiol. 203:112392. 10.1016/j.ijpsycho.2024.112392 39002638

[B78] Tan C.-S. Qu L. (2015). Stability of the positive mood effect on creativity when task switching, practice effect, and test item differences are taken into consideration. J. Creat. Behav. 49, 94–110. 10.1002/jocb.56

[B79] Terraciano A. McCrae R. R. Costa Jr P. T. (2003). Factorial and construct validity of the italian positive and negative affect schedule (PANAS). Eur. J. Psychol. Assess. 19, 131–141. 10.1027//1015-5759.19.2.131 20467578 PMC2868265

[B80] Tuccitto D. E. Giacobbi P. R. Leite W. L. (2010). The internal structure of positive and negative affect: a confirmatory factor analysis of the PANAS. Educ. Psychol. Meas. 70, 125–141. 10.1177/0013164409344522

[B81] Unkelbach C. Fiedler K. Bayer M. Stegmüller M. Danner D. (2008). Why positive information is processed faster: the density hypothesis. J. Pers. Soc. Psychol. 95, 36–49. 10.1037/0022-3514.95.1.36 18605850

[B82] van Alebeek H. Kahveci S. Rinck M. Blechert J. (2023). Touchscreen-based approach-avoidance responses to appetitive and threatening stimuli. J. Behav. Ther. Exp. Psychiatry 78:101806. 10.1016/j.jbtep.2022.101806 36435548

[B83] van Dessel P. Eder A. B. Hughes S. (2018). Mechanisms underlying effects of approach-avoidance training on stimulus evaluation. J. Exp. Psychol. Learn. Mem. Cogn. 44, 1224–1241. 10.1037/xlm0000514 29648864

[B84] Volman I. Roelofs K. Koch S. Verhagen L. Toni I. (2011). Anterior prefrontal cortex inhibition impairs control over social emotional actions. Curr. Biol. 21, 1766–1770. 10.1016/j.cub.2011.08.050 22000109

[B85] Vrijsen J. N. van Oostrom I. Speckens A. Becker E. S. Rinck M. (2013). Approach and avoidance of emotional faces in happy and sad mood. Cognit. Ther. Res. 37, 1–6. 10.1007/s10608-012-9436-9 23355753 PMC3555229

[B86] Walker W. R. Skowronski J. J. Thompson C. P. (2003). Life is pleasant–and memory helps to keep it that way! Rev. Gen. Psychol. 7, 203–210. 10.1037/1089-2680.7.2.20319229630

[B87] Watson D. Clark L. A. Tellegen A. (1988). Development and validation of brief measures of positive and negative affect: the PANAS scales. J. Pers. Soc. Psychol. 54, 1063–1070. 10.1037/0022-3514.54.6.1063 3397865

[B88] Weir P. (Director). (1989). Dead Poets Society [Film]. Burbank, CA: Touchstone Pictures; Silver Screen Partners IV; A Steven Haft Production; Witt/Thomas Productions.

[B89] Westermann R. Spies K. Stahl G. Hesse F. W. (1996). Relative effectiveness and validity of mood induction procedures: a meta-analysis. Eur. J. Soc. Psychol. 26, 557–580. 10.1002/(SICI)1099-0992(199607)26:4&lt;557::AID-EJSP769&gt;3.0.CO;2-4

[B90] Wiers C. E. Gladwin T. E. Ludwig V. U. Gröpper S. Stuke H. Gawron C. K.. (2017). Comparing three cognitive biases for alcohol cues in alcohol dependence. Alcohol Alcohol. 52, 242–248. 10.1093/alcalc/agw063 28182202

[B91] Wiers C. E. Kühn S. Javadi A. H. Korucuoglu O. Wiers R. W. Walter H.. (2013a). Automatic approach bias towards smoking cues is present in smokers but not in ex-smokers. Psychopharmacology 229, 187–197. 10.1007/s00213-013-3098-5 23604335

[B92] Wiers C. E. Stelzel C. Gawron C. Park S. Q. Gladwin T. Pawelczack S.. (2013b). Effects of approach bias modification training on neural cue reactivity in alcohol-dependent patients. Suchttherapie 14(S01). 10.1055/s-0033-135149825526597

[B93] Wiers C. E. Stelzel C. Gladwin T. E. Park S. Q. Pawelczack S. Gawron C. K.. (2015). Effects of cognitive bias modification training on neural alcohol cue reactivity in alcohol dependence. Am. J. Psychiatry 172, 335–343. 10.1176/appi.ajp.2014.13111495 25526597

[B94] Wiers C. E. Stelzel C. Park S. Q. Gawron C. K. Ludwig V. U. Gutwinski S.. (2014). Neural correlates of alcohol-approach bias in alcohol addiction: the spirit is willing but the flesh is weak for spirits. Neuropsychopharmacology 39, 688–697. 10.1038/npp.2013.252 24060832 PMC3895246

[B95] Zech H. G. Gable P. van Dijk W. W. van Dillen L. F. (2023). Test-retest reliability of a smartphone-based approach-avoidance task: effects of retest period, stimulus type, and demographics. Behav. Res. Methods 55, 2652–2668. 10.3758/s13428-022-01920-6 35915356 PMC9342838

[B96] Zhou Y. Li X. Zhang M. Zhang F. Zhu C. Shen M.. (2012). Behavioural approach tendencies to heroin-related stimuli in abstinent heroin abusers. Psychopharmacology 221, 171–176. 10.1007/s00213-011-2557-0 22113446

