# Supplementary material for: Feeling good, approaching the positive
Source: Front Psychol. 2024 Dec 18;15:1491612. doi: 10.3389/fpsyg.2024.1491612 (PMC11689285; doi:10.3389/fpsyg.2024.1491612)
Supplement: Supplementary file 1 [file Data_Sheet_1.pdf]

Supplementary Material

1 DESCRIPTIVE STATISTICS

Here, we report descriptive statistics for the relevant predictors of the preregistered analysis computed on the log-transformed and standardized data as well as the raw data in the millisecond timescale.

Table S1. Descriptive statistics for the relevant predictors of the preregistered analysis

		Mean	SD	Mean (ms)	SD (ms)
A: Main effect of AAT instruction	Congruent	-0.07	0.97	861	293
	Incongruent	0.07	1.02	900	316
B: Main effect of emotional priming	Positive	-0.01	1	876	302
	Negative	0.01	1	885	309
C: Interaction effect: AAT instruction : emotional priming	Congruent after negative emotional priming	-0.06	0.97	863	294
	Incongruent after negative emotional priming	0.09	1.03	906	322
	Congruent after positive emotional priming	-0-08	0.98	859	292
	Incongruent after positive emotional priming	0.05	1.01	894	311

2 PRIOR PREDICTIVE CHECKS

Here, we report the priors used for all models as well as perform prior predictive checks for the intercept priors and effect sizes (see Figures S1 and S2).

The priors for the reaction time models are specified as follows:

- Intercept: Normal(0, 1)
- Coefficient: Normal(0, 0.05)
- Sigma: Normal(0, 0.5)
- Standard deviation of group-level effects: Normal(0, 0.1)
- Correlation of group-level effects: LKJ(2)

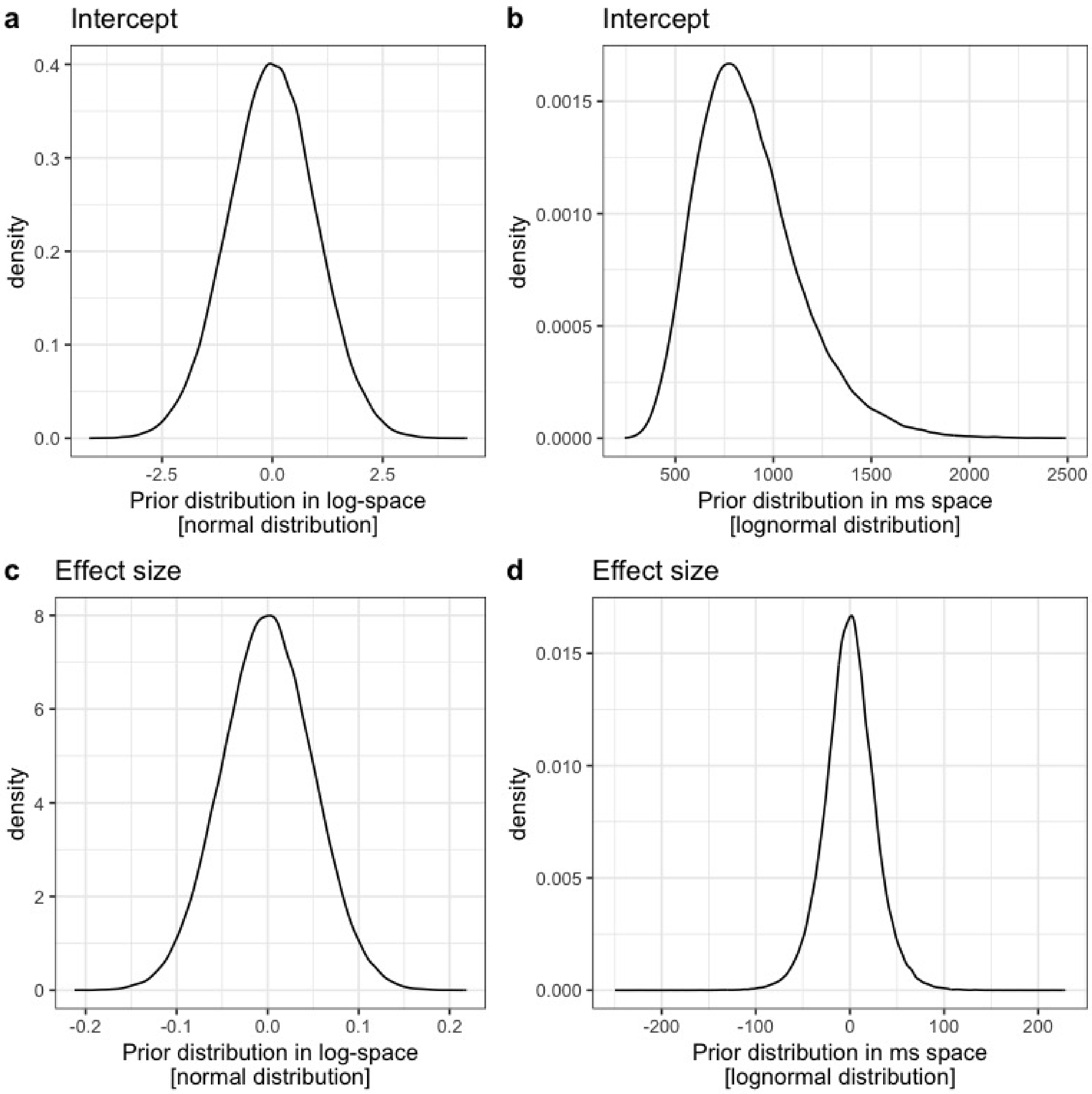

Figure S1. Prior predictive checks for the intercept prior and effect size in log-space (standardized around 0) and ms space (original data distribution) for the preregistered model.

As can be seen in Figure S1d, effect sizes ranging between -50ms and 50ms can reasonably be detected with our choice of priors. Note that the effect size we caculate here is the difference between congruent and incongruent conditions.

The priors for the PANAS model are specified as follows:

- Intercept: Normal(25, 10)
- Coefficient: Normal(0, 5)

- Sigma: Normal(0, 5)
- Standard deviation of group-level effects: Normal(0, 5)
- Correlation of group-level effects: LKJ(2)

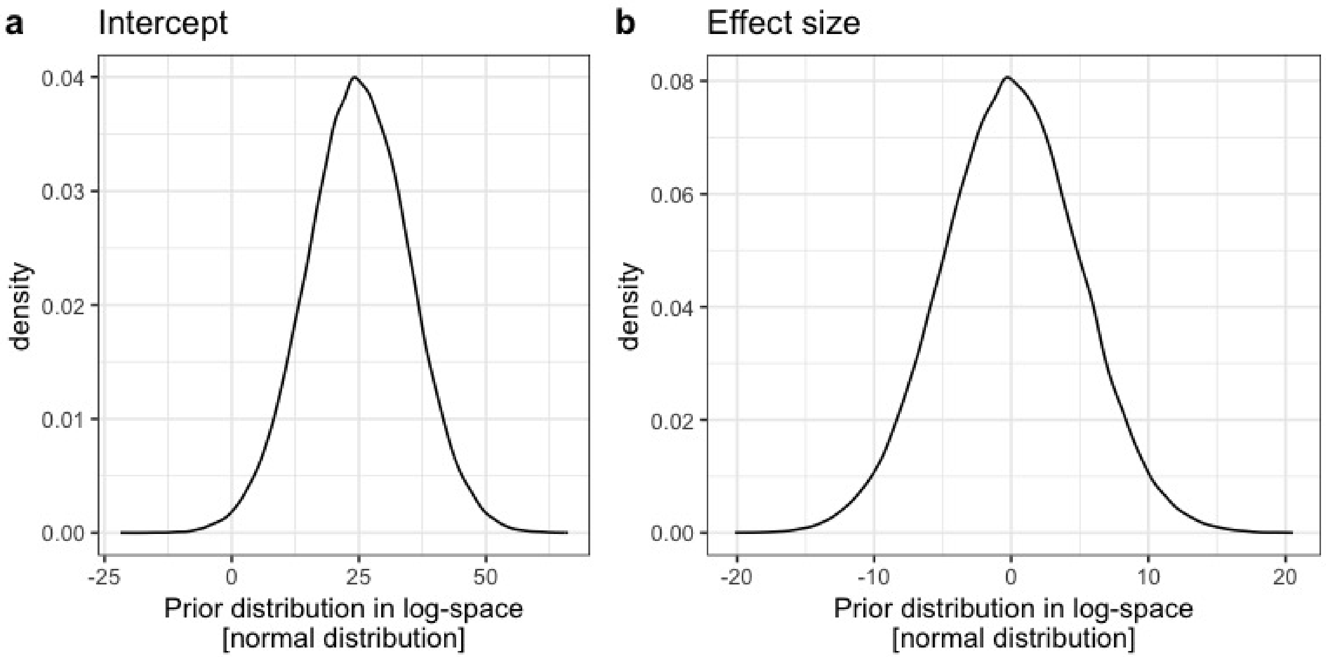

Figure S2. Prior predictive checks for the intercept prior and effect size for the PANAS model.

In Figure S2d, we show that detectable effect sizes with our choice of priors for the PANAS data range between -10 and 10 points on the PANAS scale.

3 BAYESIAN STATISTICAL MODEL OUTPUTS

Here, we report the model outputs from the Bayesian statistical models.

Table S2. Preregistered model outputs: Population-level effects

	Estimate	Est.Error	l-95% CrI	u-95% CrI	Rhat[a]	Bulk_ESS[b]	Tail_ESS[b]
Intercept	-0.03	0.06	-0.15	0.08	1.00	1,539	3,746
AAT instruction (incongruent)	0.11*	0.03	0.05	0.16	1.00	11,601	18,395
Mood induction (negative)	-0.02	0.03	-0.08	0.05	1.00	6,703	12,426
AAT instruction : Mood induction	-0.01	0.03	-0.06	0.04	1.00	17,743	22,763

[a]Rhat value must be close to 1 (< 1.05) and indicates convergence (Nicenboim et al., 2023).
[b]Effective Sample Size (ESS) should be sufficiently large ($> 1,000$, Bürkner, 2017).
**CrI does not include 0.*

Table S3. Panas Model outputs: Population-Level-Effects

	Estimate	Est. Error	l-95% CrI	u-95% CrI	Rhat[a]	Bulk ESS[b]	Tail ESS[b]
Intercept	20.50*	0.52	19.48	21.51	1.00	8,522	13,952
Mood induction (negative)	0.64	0.53	-0.39	1.67	1.00	11,547	16,744
Affect scale (negative)	0.35	0.52	-0.67	1.39	1.00	13,413	19,608
PANAS PrePost (after)	1.90*	0.42	1.07	2.73	1.00	19,262	24,004
Mood induction : Affect scale	-0.65	0.79	-2.21	0.91	1.00	12,735	18,757
Mood induction : PANAS PrePost	1.47*	0.54	0.40	2.54	1.00	21,824	24,032
Affect Scale : PANAS PrePost	-1.17*	0.53	-2.21	-0.12	1.00	22,956	23,532
Emotional Priming : Affect Scale : PANAS PrePost	0.57	0.82	-1.03	2.19	1.00	19,464	22,964

[a]Rhat value must be close to 1 (< 1.05) to indicate convergence (Nicenboim et al., 2023).
[b]Effective Sample Size (ESS) should be sufficiently large ($> 1,000$, Bürkner, 2017).
**CrI does not include 0.*

Table S4. Exploratory model outputs: Population-level effects

	Estimate	Est. Error	l-95% CrI	u-95% CrI	Rhat	Bulk_ESS	Tail_ESS
Intercept	0.00	0.06	-0.12	0.12	1.00	2,757	6,386
AAT Instruction (Incongruent)	0.08	0.03	0.02	0.13	1.00	19,009	34,049
Emotional Priming (Negative)	-0.01	0.03	-0.08	0.05	1.00	11,650	24,944
Stimulus Valence (Negative)	-0.10	0.02	-0.14	-0.06	1.00	27,541	43,738
AAT Instruction : Emotional Priming	0.04	0.03	-0.02	0.09	1.00	29,859	46,944
AAT Instruction : Stimulus Valence	0.03	0.03	-0.02	0.09	1.00	28,551	38,898
Emotional Priming : Stimulus Valence	0.06	0.03	0.01	0.12	1.00	34,956	49,105
AAT Instruction : Emotional Priming : Stimulus Valence	-0.02	0.03	-0.07	0.04	1.00	50,514	51,988

[a]Rhat value must be close to 1 (< 1.05) and indicates convergence (Nicenboim et al., 2023).
[b]Effective Sample Size (ESS) should be sufficiently large ($> 1,000$, Bürkner, 2017).
**CrI does not include 0.*

4 FURTHER ANALYSES

4.1 Effects of gender

Since our participant sample is not fully gender-balanced, we perform additional exploratory analyses where we included the participants' reported gender as a predictor in the preregistered model and in the PANAS model to rule out possible gender effects.

Table S5. Preregistered model including gender: Posterior summary statistics

	Mean	l-95% CrI	u-95% CrI	pd (in %)	% in ROPE[a]	Rhat[b]	ESS[c]
Intercept	-0.05	-0.16	0.07	79.87	29.45	1.00	1490
AAT instruction (incongruent)*	0.09	0.04	0.14	99.94	0.00	1.00	8197
Mood induction (negative)	0.01	-0.05	0.08	64.37	63.63	1.00	5153
Subject gender (male)	0.01	-0.08	0.11	62.17	47.83	1.00	18526
AAT instruction : Mood induction	0.03	-0.02	0.08	88.67	47.26	1.00	18148

[a]ROPE range: [-0.03, 0.03], i.e., about 30ms around 0.
[b]Rhat value must be close to 1 (< 1.05) and indicates convergence (Nicenboim et al., 2023).
[c]Effective Sample Size (ESS) should be sufficiently large ($> 1,000$, Bürkner, 2017).
**CrI does not include 0.*

Table S5 summarizes the model outputs of the preregistered model, including subject gender as an additional predictor. We find no effect of gender.

Table S6. PANAS model including gender: Posterior summary statistics

	Mean	l-95% CrI	u-95% CrI	pd (in %)	% in ROPE[a]	Rhat[b]	ESS[c]
Intercept*	20.36	19.32	21.44	100.00	0.00	1.00	7,024
Mood induction (negative)	0.64	-0.39	1.65	88.99	41.57	1.00	11,865
Affect scale (NA)	0.36	-0.67	1.36	75.45	62.50	1.00	12,352
PrePost (after)*	1.90	1.10	2.73	100.00	0.00	1.00	17,419
Subject gender (male)	0.65	-1.56	2.82	72.19	32.97	1.00	10,178
Mood induction : Affect scale	-0.65	-2.17	0.87	79.82	39.72	1.00	11,929
Mood induction : PrePost*	1.47	0.39	2.51	99.61	1.88	1.00	19,110
Affect scale : PrePost*	-1.17	-2.19	-0.11	98.76	9.21	1.00	19,996
Mood induction : Item affect : PrePost	0.58	-0.95	2.24	76.04	41.55	1.00	17,162

[a]ROPE range: [-0.03, 0.03], i.e., about 30ms around 0.
[b]Rhat value must be close to 1 (< 1.05) and indicates convergence (Nicenboim et al., 2023).
[c]Effective Sample Size (ESS) should be sufficiently large ($> 1,000$, Bürkner, 2017).
**CrI does not include 0.*

Table S6 summarizes the results of our PANAS model including gender as a main effect. We find no effect of gender.

4.2 PCA analysis of PANAS items

As for example suggested by Ekkekakis and Zenko (2016), the PANAS questionnaire mixes together items belonging to various affective categories such as mood, emotion and core affect and items belonging to either high- or low-activation states. Therefore, we tried to identify subscales on the PANAS questionnaire that we could use for a more fine-grained subscale analysis. Because no full list of which item on the PANAS belongs to which category could be retrieved from the literature, we decided to perform a Principle Components Analysis (PCA) to detect subscales directly from the data structure.

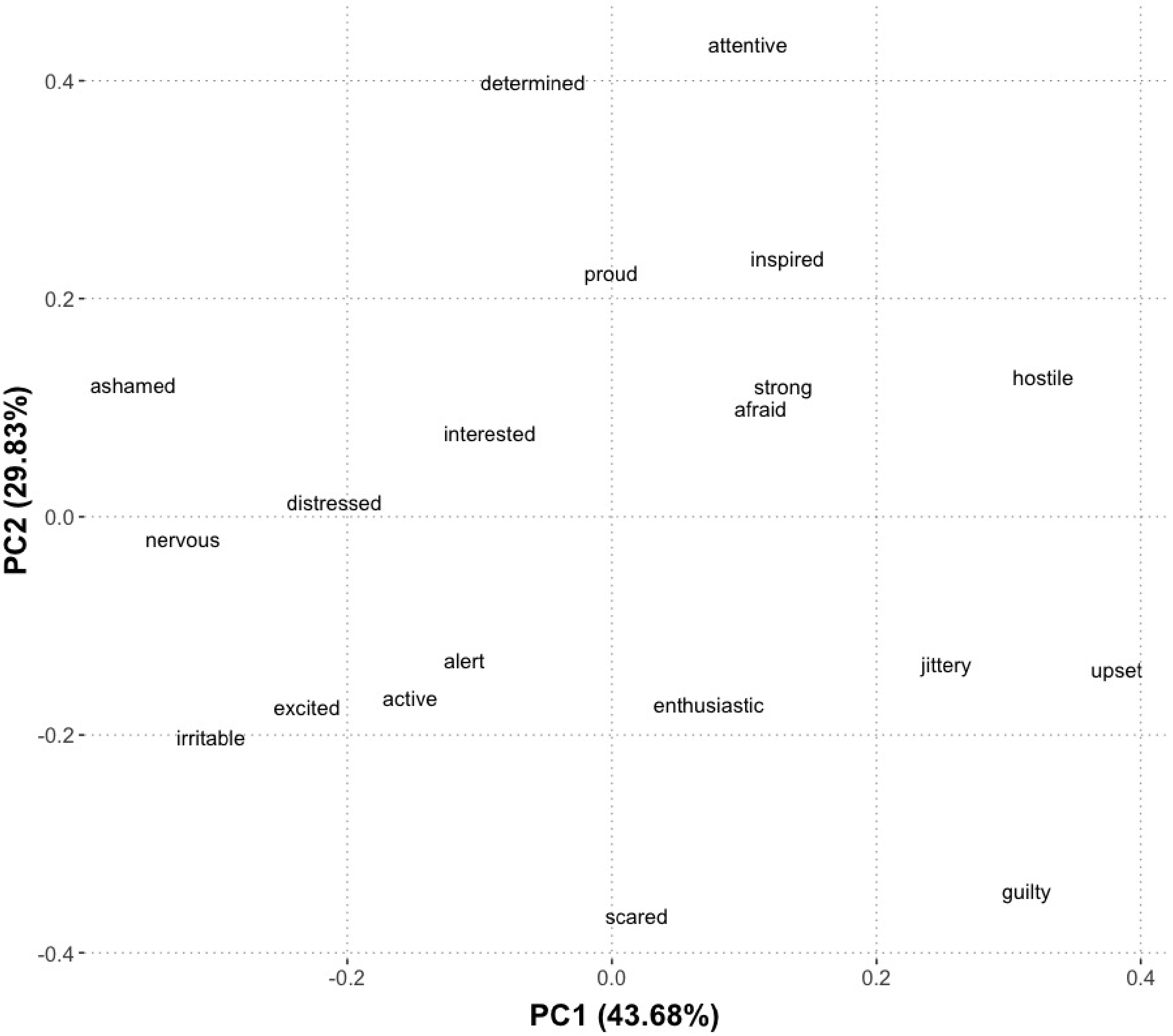

Figure S3. Principle components of the PANAS items

Figure S3 plots the two components that explain the highest variance in the data (PC1 and PC2) on the x- and y-axis. We observe that the PANAS items are scattered in this space, and no clusters can be identified. We conclude that, expect for positive and negative affect, subscales in the PANAS questionnaire are mixed

and cannot be disentangled. If the goal is to disentangle the two affective dimensions of valence and arousal, a scale should be constructed in future research that explicitly includes the dimension of arousal.

REFERENCES

Bürkner, P.-C. (2017). brms: An R package for Bayesian multilevel models using Stan. *Journal of Statistical Software* 80, 1–28. doi:10.18637/jss.v080.i01

Ekkekakis, P. and Zenko, Z. (2016). Measurement of affective responses to exercise: From "affectless arousal" to "the most well-characterized" relationship between the body and affect. In *Emotion Measurement*, ed. H. L. Meiselman (Woodhead Publishing). 299–321. doi:10.1016/B978-0-08-100508-8.00012-6

Nicenboim, B., Schad, D., and Vasishth, S. (2023). *An Introduction to Bayesian Data Analysis for Cognitive Science* (https://vasishth.github.io/bayescogsci/book/)
